# ABSTRACTS OF THE WORKS PRESENTED AT THE 16^th^ Academic Conference of Speech-Language Pathology and Audiology of the Bauru School of Dentistry “Dr. Kátia de Freitas Alvarenga”

**DOI:** 10.1590/S1678-77572009000700023

**Published:** 2009

**Authors:** Giédre Berretin-Félix, Magali de Lourdes Caldana, Nicolle Carvalho Sant'Ana

## AUDIOLOGY

### A001 Auditory processing in unilateral hearing loss: case report

Salvador, Karina Krähembühl^1^ – ka_salvador@yahoo.com.br

Duarte, Tâmyne Ferreira^1^

Feniman, Mariza Ribeiro^1^

^1^Bauru School of Dentistry - University of São Paulo.

The unilateral hearing loss has been studied by a large number of researchers because of damages that it can bring to central nervous system's maturation, mainly when it refers to the development of the binaural abilities: sound localization, sequential auditory memory, selectivity attention, discrimination, perception in the presence of competing background of noise; considering that one ear probably completes and/or aid the other in the process of decoding the auditory signals. Therefore, the aim of this paper was verify the central auditory abilities in a male individual, 17 years old, with the diagnosis of profound unilateral sensorineural hearing loss and idiopathic cause, without other endangers. The process of evaluation consisted of the application of a questionnaire, conventional audiologic clinical evaluation (audiometry, speech audiometry and timpanometry) and auditory processing tests, being applied to the patient in this study only those classified as monotics and diotics. We concluded that an individual with profound unilateral sensorineural hearing loss could obtain the same scores pre-established in central tests that are standard for individuals with normal binaural hearing. Nevertheless, the binaural stimulation brings greater benefits for development of central auditory abilities, influencing its functional aspect. This last parameter can be ascertained by the incompatibility between the scores obtained in the central tests and the patients' complaint related to their routine.

### A002 Auditory monitoring in infants in the first year of life

Giordano, Bianca Celestino^1^ – biagiordano@yahoo.com.br

Colella-Santos, Maria Francisca^1^

^1^School of Medical Sciences State University of Campinas – UNICAMP.

The auditory system is extremely important to infants, with regard to their overall development. Hence, early detection of possible compromised hearing is essential in order to provide stimulation and an appropriate intervention. Therefore, the objective of this study was to analyze the auditory responses of infants at 4, 8 and 12 months of age, which have shown normal results in neonatal hearing screening, but who have risk indicators for late and/or progressive onset hearing loss. For such purpose, the infants born in CAISM/Unicamp and State Hospital of Sumaré, who were approved in hearing screening, but remained hospitalized in the ICU, and those who had risk indicators for late and/or progressive onset hearing loss, were referred to the completion of hearing monitoring. The following procedures have been performed: anamnesis; behavioral observation with verbal and non-verbal sound; visual reinforcement audiometry (PA2, of Interacoustics); immittance (middle ear analyzers MT10, of Interacoustics); conduction of study of otoacoustic emissions (ILO 292 USB, of Otodynamics). These different methods were chosen according to the age of the infant. Sixty-five infants were evaluated (36 males and 29 females). Regarding the gestational age, 53.85% are pre-terms. Delay in auditory development was verified in 37.2% of infants of four months of age, 50% of infants of 8 months and 27.3% of infants of 12 months. The immittance presented variations in 23.5% of infants of four months of age, 33.3% of infants of eight months, and 9.1% at 12 months. For children who had responses under expectation, explanatory leaflets were given to their parents with suggestions for activities to be developed at home in order to stimulate the hearing. Therefore, the importance of carrying out this monitoring is understood, as possible hearing loss or even late auditory development might be early diagnosed.

### A003 Audiological findings in patients with facial paralysis

Ortolan, Natália da Conceição Rossi^1^ – natalia_ortolan@terra.com.br

Silva, Daniela Polo Camargo da^1^

Fioravanti, Marisa Portes^1^

Tamashiro, Ivanira Ayako^1^

^1^School of Medicine of Botucatu – UNESP.

The peripheral facial paralysis is the result of the interruption of the nervous influx of any segments of the facial nerve. Its involvement results in partial or complete paralysis of the facial movements and may be associated with disturbances of taste, dribble, and tears, in addition to changes in speech, chewing, swallowing and sucking. In 50% of cases, the etiology is unknown. The first incidence is idiopathic, or Bell's paralysis, and the second is traumatic. Symptoms such as tinnitus, vertigo and hearing loss may be associated, especially in cases of tumor. Eighteen individuals with idiopathic facial palsy participated in the study, 10 males and 8 females, mean age of 37 years. All of them underwent medical evaluation and then were referred for audiological assessment that included tests of pure tone audiometry (PTA) and impedanciometry. PTA in six ears had mild hearing loss compatible with speech audiometry. The downward curve was observed in 56% of the ears examined. In impedanciometry, the tympanometric type A curve and the absence of reflex were the most common. This study showed hearing loss and mild downward curve in subjects presented with idiopathic facial paralysis. The presence of decruitment was also observed. The audiometry, in these cases, should be performed to verify the presence of deafness, especially in the speech frequencies, and together with the impedanciometry may help in the diagnosis of peripheral facial paralysis.

### A004 Tinnitus and audiometric findings

Ortolan, Natália da Conceição Rossi^1^ – natalia_ortolan@terra.com.br

Silva, Daniela Polo Camargo da^1^

Fioravanti, Marisa Portes^1^

Tamashiro, Ivanira Ayako^1^

^1^School of Medicine of Botucatu – UNESP

Tinnitus is one of three major otoneurological events besides vertigo and sensorineural hearing loss. It can be generated by sounds of structures near the inner ear and transmitted to the cochlea or occur in situations in which the dysfunction is in somewhere in the auditory system, from the neuroepithelium structures of the Corti's organ to the auditory cortex. The latter type is often associated with hearing loss as in cases of acoustic trauma, use of ototoxic drugs, Presbyacusis, Menière's disease and in individuals with vestibular schwannoma. Tinnitus can be mentioned in one or both ears, or inside the head. Many people may have tinnitus before they show some degree of deafness. Ninety-five patients with mean age 50 years, of both genders, were submitted to pure tone audiometry (PTA), after medical evaluation. Sixty-five percent with complaint of tinnitus only and 35% with tinnitus and deafness, and 32% unilaterally and 68% bilaterally. Of those who had only complained of tinnitus, 29% had hearing loss with mild degree and a descending curve. Thus, the hearing health monitoring in individuals with tinnitus, without complaint of deafness, is relevant for a better treatment and assistance of the progression of the disease which is causing these symptoms.

### A005 Audiological findings in patients with sudden deafness

Ortolan, Natália da Conceição Rossi^1^ – natalia_ortolan@terra.com.br

Silva, Daniela Polo Camargo da^1^

Fioravanti, Marisa Portes^1^

Tamashiro, Ivanira Ayako^1^

^1^School of Medicine of Botucatu – UNESP.

Sudden deafness is characterized by a sudden onset of hearing loss with varied etiology. The deafness may develop in hours or days, usually unilateral, with variable intensity and may be mild to profound. In many cases, tinnitus (80%) and vertigo (30%) may be associated. Among the causes of sudden deafness are the viruses, the vascular disturbances, changes in barometric pressure, the acoustic trauma, head trauma and the vestibular schwannoma. Sudden deafness may be permanent, but may also have a spontaneous recovery of normal hearing level or close to normal. Its incidence is similar between men and women. The age of patients ranges from 40 to 60 years. Thirty-four subjects with complaints of sudden deafness, 16 females and 18 males with a mean age of 39 years, participated of this study. Most participants showed unilateral complaint accompanied by tinnitus. All of them underwent the medical evaluation and then were referred for audiological assessment that included tests of pure tone audiometry (PTA) and impedanciometry. In the medical evaluation, 10 ears presented changes and 58 normal otoscopy. PTA in 65% of ears showed some degree of hearing loss and the mild degree and descendent curve were the most common. In speech audiometry, most patients showed discrimination compatible with the degree of deafness. In the impedanciometry, the tympanometric type A curve was the most frequent, and nine ears had recruitment. This study showed that sudden deafness causes varying degrees of hearing loss, being the mild one the most common. The etiology of this type of loss should be carefully investigated for appropriate intervention and imaging tests are often needed to clarify the diagnosis in cases of vestibular schwannoma.

### A006 Occupational hearing loss: record of the audiometries performed in USP Bauru Campus

Otubo, Karina Aki^1^ – kari.otubo@usp.br

Lopes, Andréa Cintra^1^

Basso, Talita Costa^1^

Marinelli, Érica Juliana Innocenti^1^

Macedo, Camila^1^

Lauris, José Roberto Pereira^1^

^1^Bauru School of Dentistry - University of São Paulo.

Studies on occupational exposure demonstrate that noise has been reaching a great part of the working population, worldwide, being the Noise Induced Hearing Loss (NIHL), the second most frequent disease of the auditory system. The exposure to intense noise, for long periods causes auditory effects, such as temporary or permanent hearing loss and acoustic trauma, as well as non-auditory ones (tinnitus, vertigo, amongst others). Aiming at enlightening the new findings and improving the auditory quality of the workers, the main objective of this study was to analyze the audiometries of the employees of USP, Campus at Bauru, in environments whose sound pressure level exceeded 85 dBNPS. Using a retrospective model, two groups of participants exposed to occupational were analyzed: those with tonal thresholds within the acceptable limits and those who presented alterations in the hearing thresholds, that is, tonal thresholds below 25 dB, in any frequency (Decree number 19 of the Ministry of Work and Employment −1998). Forty periodic audiometries carried out between 2007 and 2008, from individuals of both genders aged 32 to 59 years, and with varied professions (gardeners, maintenance technicians, drivers, among others) were analyzed. According to the classification proposed by Fiorini (1994), 27.5% (N=11) presented audiometries within normality, 45.0% (N=18) presented normal audiometries with notch; and 25.0% (N= 10) presented audiometric configuration suggestive of NIHL. Besides the Conventional Audiological Evaluation (from 250 to 8,000 Hz), the High Frequency Audiometry was also performed (9,000Hz, 10,000Hz, 11,200Hz, 12,500Hz, 14,000Hz and 16,000Hz). The results showed that the two studied groups presented worse responses in the High Frequency Audiometry, demonstrating that the use of this registry seems to be useful as a method for early detection of auditory alterations.

### A007 Elderly people, new users of hearing aids: expectations and satisfactions

Santos, Izabella dos^1^ – izabella.santos@terra.com.br

Couto, Christiane Marques do^1^

^1^School of Medical Sciences State University of Campinas – UNICAMP.

Aging causes changes in hearing and may lead elderly people to communication difficulties. So, many of them look for the hearing aids to solve their difficulty to hear and their psycho-social problems. However, elderly people have expectations that may influence their adaptation to the hearing aids. This research examined the relationship between their expectations and their satisfaction. Data collection was done in two phases: the first was when the elderly people received their hearing aids and the second, a month after. A semi-open questionnaire, formulated by the researcher, was applied and estimated their expectation in the first and the second phases. In the second phase of the study, the questionnaire termed “International Outcome Inventory for Hearing Aids (IOI-HA)”, developed by Cox (2002) was also applied. This questionnaire analyses the satisfaction and other aspects, regarding the use of hearing aids. People between 60 and 85 years old, with sensorineural hearing loss, belonging to the Hearing Health Program at UNICAMP, participated in the study. The first phase of the research involved 14 people, and the second phase, 10 out of the 14, from the initial phase. It was observed that most elderly people expected that the hearing aids would improve their communication with the family, in their leisure or at work. Issues related to esthetics emerged among the female participants. In the second phase, it was observed that they determined better their speaker or the object they desired to hear better. As for the responses to the questionnaire IOI-HA, 70% of elderly people had a score between 29 and 35. The study showed that expectations become more specific after use of hearing aids and that most participants evaluated the hearing aids positively, being satisfied with their better hearing after their use.

### A008 Amplified music and adolescents: where does the danger live?

Sant'Ana, Nicolle Carvalho^1^ – nicolle.santana@gmail.com

Lopes, Andréa Cintra^1^

Fernandes, Gabriela^1^

^1^Bauru School of Dentistry - University of São Paulo.

Nowadays the use of individual amplified music is very different from the past due to the larger space for storage and the long battery duration, which contributes to users listening to music during hours without brakes, often in a volume that would not be advisable, allowing more early hearing damage. The study aimed to promote educational lectures, with hearing health promotion in elementary, middle and high schools, in order to minimize early hearing damages. Two schools participated in the study, one public and one private, which included adolescents between 11 to 18 years old, listeners or not of amplified music. The material was exposed in slides, pictures and videos and approached themes about hearing, use of amplified music electronic devices, hearing health risks, hearing and not hearing symptoms, among other information. The results showed the participation of all present students during the lecture, with questions and comments that emphasized that the youth has litle knowledge of hearing loss and the damages it may cause, making indiscriminate use individual amplified music devices. Therefore, it is necessary to continue hearing health programs in this population, with hearing health promotion and early diagnosis tests to ensure that these young adults are protected against the potential harmful effects, in benefit of their present and future health.

### A009 Implications of neurosarcoidosis in audiology: case report

Oliveira, Jerusa Roberta Massola de^1^

Manoel, Rosana Ribeiro^1^

Blasca, Wanderléia Quinhoneiro^2^

Campos, Karis de^2^ – karisdecampos@yahoo.com.br

^1^Hearing Division of Health, Hospital for Rehabilitation of Anomalies Craniofaciais-HRAC/USP.

^2^Bauru School of Dentistry - University of São Paulo.

Sarcoidosis is a multiorganic granulomatous disease that can affect any organ or system. Research has shown higher incidence in the lungs and intrathoracic lymph nodes, and therefore this condition has diverse clinical manifestations. The VIII cranial nerve can be affected with consequent sensorineural hearing loss. Anyone can contract the disease, noting that 70% of patients with this disease are less than 40 years old. Its etiology remains unknown and in many cases the diagnosis is established by exclusion. This study aimed to describe the clinical and audiological findings in a 16-year-old female patient with neurosarcoidosis enrolled in CEDALVI the HRAC USP, where the audiological diagnosis and the procedures for selection of hearing aids were performed. Data were collected from the patient's mother report, results of tests performed since the time the first symptoms had manifested, in addition to analysis of medical records with a focus on speech anamnesis, ENT, nursing assessment and audiological aspects as pure tone audiometry and acoustic impedance measurements. Clinical symptoms and neurological problems that occurred were more headaches, facial paralysis, tinnitus, and hearing impairment as sequelae of the sensory neural type of severe degree on the left ear and profound level on the right ear. This study shows the importance of research in auditory function in individuals with neurosarcoidosis.

### A010 Relation between hearing handicap and audiometric data

Aiello, Camila Piccini^1^ – mi.aiello@gmail.com

Lima, Ivanildo Inácio de^1^

Ferrari, Deborah Viviane^1^

^1^Bauru School of Dentistry - University of São Paulo.

Traditionally the selection of adult candidates for an aural rehabilitation program is based only on audiometric criteria. However, other factors influence the emotional and/or functional adjustments of an individual towards a hearing impairment. This study verified the relations between the self-perceived hearing handicap (participation restriction) and average audiometric thresholds as well as the speech recognition thresholds (SRT) in hearing impaired adults. Seventy seven adults (mean age: 50 years) with post lingual sensorineural bilateral hearing loss of varying degrees were evaluated. None of the participants had previous experience with hearing aid use. The Hearing Handicap Inventory for Adults (HHIA) was administered in an interview format right after the auditory diagnosis process. The inventory is comprised of two subscales exploring both the emotional (13 items) as well as the social/situational (12 items) consequences of hearing impairment. Pearson's correlation coefficients between HHIA scores (total as well as the emotional and social subscales scores) and audiometric thresholds (ISO average, encompassing the frequencies of 500, 1k, 2k and 4 kHz) and SRT values of the ear with better residual hearing were calculated. A significance level of 5% was adopted. Very weak correlations were found between HHIA scores and audiometric thresholds and SRT, this being statistically significant in case of audiometric thresholds. These results reinforce the need for the use of an instrument for evaluating participation restriction in clinical practice since it cannot be inferred from the audiometric data alone.

### A011 Selective and sustained auditory attention in children with cleft lip and palate

Salvador, Karina Krähembühl^1^ – ka_salvador@yahoo.com.br

Duarte, Tâmyne Ferreira^1^

Zotelli, Camila Monteiro^2^

Camargo, Renata Arruda^2^

Feniman, Mariza Ribeiro^1^

Carvalho, Fernanda Ribeiro^3^

^1^Bauru School of Dentistry - University of São Paulo.

^2^Hospital for Rehabilitation of Craniofacial Anomalies – HRAC.

^3^University of Sacred Heart.

Auditory attention is the ability one has to focus on a sound stimulus and is ready to receive a different stimulus. It is essential to acquire acoustic and phonetic aspects of language patterns which are imperative to the learning process. Selective and Auditory attention are outlined in this study. Auditory selective attention involves answering an acoustic stimulus over another, so that the reaction is directed to a significant stimulus ignoring the other one. In Pediatric Test of the Speech Intelligibility - PSI, the task involves selective attention. Auditory sustained attention refers to the process of focusing on a particular stimulus during a period of time. This ability is tested using the Sustained Auditory Attention Ability Test (SAAAT). Given that cleft lip and palate is a risk indicator for hearing and that middle ear alterations may provide long periods of sensorial deprivation changing auditory abilities, the aim of this study was to evaluate whether there is correlation between the results of PSI and SAAAT in children with cleft lip and palate. 40 medical records of children with cleft lip and palate, aged 7 to 11 years, from a Craniofacial Malformation Hospital, with normal hearing were evaluated in a retrospective study. 32.5% of cases in PSI test showed alterations. 69.23% of these also had variation in SAAAT. Of the children who showed no change in PSI (67.5%), 48.15% showed alterations in SAAAT. There was no statistical correlation among the results. Thus, according to the results, alterations in auditory sustained attention are not related to alterations in auditory selective attention. However, further investigation on auditory sustained and selective attention is needed.

### A012 Use of auditive prosthesis and satisfaction

Nam, Tatiana My Reom^1^ – dandara1@uol.com.br

Couto, Christiane Marques do^1^

^1^School of Medical Sciences State University of Campinas – UNICAMP.

Factors that contribute to use of hearing aids: acceptance, satisfaction, benefit. Satisfaction does not depend only on acceptance and benefit, but includes the individual's expectances regarding the prosthesis. This study analyzed satisfaction, period of prosthesis use and relationship between them. The International Outcomes Inventory - Hearing Aids (IOI-HA) was applied, which investigates seven dimensions of hearing aids: daily use, benefit, residual activity limitations, satisfaction, residual participation restrictions, impact on others, quality of life and a questionnaire on time of use prepared for this research, which investigated situations, reasons for use and non-use. In IOI-HA, highest score evaluation becomes more positive. Thirty people with sensoneurorial hearing losses with minimum of three months of hearing aid use participated in the research. The participants were aged 30 to 92 years, 18 males (mean age of 69.47 years) and 12 females (mean age of 45.75 years). Sample was divided in two groups, according to the difficulty classification without prosthesis (Question 8 – IOI-HA). Group 1 with an mean age of 61.76 years, judged as moderately severe hearing impairment to severe. Group 2, with mean age of 67.22 years, judged difficulty hearing as mild or moderate. We observed that positive evaluation of prosthesis, period of prosthesis use, and satisfaction are not related to judgment of hearing difficulty. We didn't observe correlation among IOI-HA variables, and among those variables and specific using time. We concluded that there is satisfaction of individuals regarding the use of prosthesis and a long time of use, but there was no statistically significant correlation between satisfaction and length of use of hearing aids, and there was no difference between satisfaction and length of use of hearing aids among the groups.

### A013 Evaluation of satisfaction of users of individual sound amplification equipment

Magalhães, Fabiani Figueiredo ^1^ – figueiredomagalhaes@uol.com.br^1^

Mondelli, Maria Fernanda Capoani Garcia^1^

Jacob, Regina Tangerino Souza^1^

^1^Bauru School of Dentistry - University of São Paulo.

Hearing is one of the fundamental senses of life, has an important role in society and is the basis for the development of human communication. Problems resulting from hearing loss can be minimized with the use of hearing aids. There are some cases of motivation that must be taken into account during the orientation of the users: how to accept the benefit and satisfaction. Satisfaction is built to the subjective impressions of the individual. However, it is essential to verify the level of satisfaction among users of hearing aids through the application of the questionnaire Satisfaction with Amplification in Daily Life (SADL). Form of study: systematic review. It was performed a search of medical literature in the LILACS and MEDLINE databases, from 1996 to 2009, Google Scholar, and Scielo Cohrane. The abstracts and articles identified by electronic search were analyzed conducted that study with the questionnaire (SADL). In the electronic search, the terms hearing, hearing aids, satisfaction, job satisfaction, and effectiveness questionnaires, were used alone and in combination. Subsequently, the data indicating that the questionnaire (SADL) was useful for evaluating the effectiveness and satisfaction of individual users of hearing aid were extracted. SADL was proven an appropriate tool to estimate the satisfaction with hearing aids to be practical, be returned to the clinical use and to allow the measurement of satisfaction in a subjective way of individuals. There was high satisfaction with the hearing in all areas of SADL. This study showed good performance to identify the effectiveness of the questionnaire (SADL) in users of hearing aid. The results also warned to the need of more research using the questionnaire, due to the lack of studies on the subject.

### A014 Audiological evaluation in patients with acquired hypothyroidism

Santos, Karlos Thiago Pinheiros dos^1^ – karlosthiago@gmail.com

Amorim, Raquel Beltrão^2^

Martins, Regina Helena Garcia^1^

^1^School of Medicine of Botucatu – UNESP.

^2^Bauru School of Dentistry - University of São Paulo.

Authors have reported an incidence around 25% of hearing loss in hypothyroidism. The physiopathology, risk cofactors, and lesion sites are still unclear. The objective of this study was to evaluate the hearing acuity, cochleovestibular symptoms, and risk cofactors in hypothyroidism. The experimental group (GA n-30) had patients with acquired hypothyroidism, and GC (n=30) had controls. Parameters: age, gender, time of diagnosis, comorbidities, cochleovestibular symptoms, biochemical and hormonal exams, audiometry, BERA and TOAEs. All participants were female. The predominant age range in both groups was 31 to 50 years. Most GA patients had less than 5 years hypothyroidism diagnosis. The most common GA comorbidities were depression and hypertension. Cochleovestibular symptoms were more frequent in GA (76.7% versus 26.7%). Mild elevation of fasting glycemia values occurred in 40% GA and 10% GC; elevated triglyceride and cholesterol levels were observed in both groups. Altered audiometry was found in 22 GA and 7 GC ears showing mild neurosensorial hearing loss. BERA was altered in 10 GA ears (eight of which also had altered audiometry), mainly prolonged LAV. TOAEs was absent in 12 GA and 4 GC ears. Cochleovestibular symptoms and alterations in the audiometry, BERA and TOAEs exams were more frequent in hypothyroidism patients.

### A015 Incidence of vestibular alterations of the dentists in the city of Manaus-AM

Carvalho, José Luiz Brito de^1^ – jluizbrito25@hotmail.com

Korbes, Neodete^2^

Alcantara, Thelma Paranhos Lima^2^

Silva, Andréa Cordeiro da^2^

Souza, Andréia Kelly Assis de

Santos, Danielle Braga dos

^1^Bauru School of Dentistry - University of São Paulo.

^2^University Center North.

The vestibular apparel is the main peripheral organ of the balance and posture. The otoneurology studies the hearing and vestibular system of the internal ear. The objective of this study was to investigate the incidence of vestibular alterations in dentists of the city of Manaus-AM. Sixty dentists aged 30 to 59 years were enrolled. Only professionals with minimum of 5 years of professional experience were selected. The data were collected in April 2008 by means of a questionnaire with closed questions. The results showed that: regarding the professional experience, 21 (35%) had 5 to 10 years, 9 (15%) 11 to 15 years, 13 (21.7%) 31 to 35 years; regarding to complaints of corporal unbalance, it was reported by 18 (30%) interviewees, being constant in only 1 (1.7%);regarding the period of daily practice, 5 (8.3%) worked 1 shift, 33 (55%) 2 shifts and 22 (35.7%) 3 shifts; 15 (25%) of the interviewed dentists referred cases of hearing losses in the family. The most frequent otoneurogical complaint was dizziness (30%). Taking into account the non-hearing effects, the most frequent complaint was headache (50%), stress (48.3%), irritability (45%), tiredness (41.7%), anxiety (36.7%) insomnia (25%), mood alterations (23.3%), variation of memory (21.7%), which can be indicative of hearing loss caused by high levels of sonorous pressure. These outcomes can be quite concerning because they are related to professional life, and can cause different physical, mental and social damages to the dentist.

### A016 Characterization of the Wave V latency-intensity function in the search for the electrophysiological threshold, according to the age group

Godoy, Juliana Fernandes^1^ – godoy.juliana@gmail.com

Von Saltiél, Débora^1^

Amorim, Raquel Beltrão^1^

Agostinho-Pesse, Raquel Sampaio^1^

Alvarenga, Kátia de Freitas^1^

^1^Bauru School of Dentistry - University of São Paulo.

The maturation process of the brainstem auditory ways, which intensifies after birth in both full-term newborns and in preterm infants, generates significant changes in wave latencies of the auditory brainstem response (ABR), for they are predominantly determined by myelinization of axons and maturation from synaptic mechanisms. By the end of the second year of life, around 18 months in children born at term, the maturational process of the auditory nerve and brainstem is complete. The research of ABR in infantile audiological evaluation is of paramount importance, especially in children under six months old, when there are no procedures to determine the psychoacoustic threshold. The objective of this study was to determine the wave V latency-intensity function observed in the definition of the electrophysiological threshold by means of ABR research, according to age. We analyzed the results in 62 ears of children born at term with no change in the external ear and / or medium and with absence of neurological diseases, age between zero and 11 months of life. The research of the electrophysiological threshold started in 80 dBHL, decreasing every 20 dBHL, to determine the last intensity in which wave V was recorded. The electrophysiological threshold was of 25.56±7.94, being found in 20 dBHL regardless the age. The latency of wave V in the intensity of 80 dBHL decreased with age, showing the maturational process from the brainstem. In the threshold research, the wave V latency was of 6.58±0.35 ms in 80 dBHL, 7.0±0.39 ms in 60 dBHL, 7.79±0.56 ms in 40 dBHL and 8.71±0.71 ms in 20 dBHL. Given the subjectivity in the analysis of ABR, it is important to know and consider both the age and intensity for adequate analysis of the ABR and consequently in the definition of the electrophysiological threshold.

### A017 Incidence of hearing loss in a neonatal auditory health program

Agostinho-Pesse, Raquel Sampaio^1^ – raquelagostinho@usp.br

Amorim, Raquel Beltrão^1^

Alvarenga, Kátia de Freitas^1^

^1^Bauru School of Dentistry - University of São Paulo.

Programs for early identification and intervention have been developed in various regions of Brazil. The literature describes the incidence of hearing loss among neonates as being 1 to 3 in every 1,000 births, and about 2 to 4, in every 1,000 from the Intensive Care Unit. The aim of this study was to determine the incidence of hearing loss in newborns, correlating with the presence or absence of risk indicators for hearing loss. It was a retrospective study from October 2003 to November 2008, which analyzed the medical records of newborns with the hearing screening program of the “Santa Isabel Maternity”, in the city of Bauru/SP, by the Auditory and Speech Pathology Department of the Bauru School University of São Paulo, campus at Bauru, which is inserted in the Newborn Hearing Health Model Project. During this period, there were 20,251 births with 134 deaths, and therefore, 20,117 newborns should be screened. Out of this total, 16,962 (84.31%) newborns were submitted to hearing screening, with 16.451 (96.98%) with the result PASSing while 511 (3.01%) FAILED, being referred to the diagnostic process to verify their hearing impairment. Out of the newborns who were submitted to the hearing screening, 1,903 (11.21%) had one or more risk indicators, of which 1,702 (89.43%) passed the newborn hearing screening (NHS), 125 (6.56%) failed and 76 (3.99%) did not complete the NHS. The most commonly found risk indicators were prematurity, followed by family history of HL, phototherapy, permanence in the intensive care unit for over than 48 hours, use of ototoxic drugs, mechanical ventilation for more than 5 days, low weight, low Apgar score, congenital infections during pregnancy, syndromes associated with hearing loss, craniofacial anomalies, hyperbilirubinemia at blood exchange transfusion and only one case of bacterial meningitis. The hearing loss was confirmed in 14 cases, 11 with the presence of risk indicators. These data confirm that there is a significant association between neonates who have risk indicators and the presence of hearing loss.

### A018 Audiometric profile of patients with tinnitus complaint

Bakr, Aline Ahmad^1^ – li.bakr@gmail.com

Freitas, Thaís Domingues^1^

Cardoso, Ana Claudia Vieira^1^

^1^School of Philosophy and Sciences of Marília – UNESP.

Tinnitus has been defined as the conscientious perception of a sound that originates in the ears or the head of the patient, without the presence of a generating external source of this sound. It is a very frequent symptom, affecting 15% of the Americans, according to National Institute of Health (1996). Many patients with tinnitus complaint present associated hearing loss, and the knowledge of this symptom is basic for the medical diagnosis of the case. The objective of this study was to characterize the audiological findings of patients who presented with tinnitus complaint. Forty-two patients of both genders with mean age of 53 years who attended the CEES/Unesp for audiological evaluation were examined. We made pure tone air and bone conduction in acoustics cabin, using audiometer and evaluated the sonorous frequencies of 250 to 8,000 Hz. The auditory thresholds were classified in accordance with Davis and Silvermann, 1970. Of the 42 evaluated patients we diagnosed: 20 (47.6%) with auditory thresholds within the normal limits; 17 (40.4%) with bilateral sensorineural hearing loss, being 9 (21.4%) symmetrical and 8 (19%) asymmetrical, and 5 (12%) with unilateral sensorineural hearing loss. Our findings allowed us to prove that patients with tinnitus complaint present associated hearing loss.

### A019 The importance of the nursing team in the newborn hearing screening program

Libardi, Ana Lívia^1^ – alibardi@yahoo.com.br

Carvalho, José Luiz Brito de^1^

Neves, Thaíla Affonso Pimenta^1^

Agostinho-Pesse, Raquel Sampaio^1^

Beltrão, Raquel Amorim^1^

Alvarenga, Kátia de Freitas^1^

^1^Bauru School of Dentistry - University of São Paulo.

The Newborn Hearing Screening Program (NHSP) is very important in the identification of hearing alterations in the first months of life. For an effective program, it is fundamental the knowledge and the valorization on the part of all health professionals involved in the gestation period and in the health of the newborn. In the NHSP of the Santa Isabel Maternity, of the city of Bauru, the hearing screening should be accomplished before the maternity discharge, and when it is not possible, through appointment with the nurse during the orientation in the moment of the maternity discharge. The objective of this study was to describe the importance of the nursing in the NHSP. The newborn's records were analyzed in the Santa Isabel Maternity, between March and August of 2008. During this period, 1,980 newborns should be screened, however 1,739 (87.8%) newborns were actually submitted to hearing screening. Of these, 828 newborns were submitted to the hearing screening before the maternity discharge (47.3%) and 1.153 (52.4%) were scheduled for hearing screening. Out of the latter sample, 911 newborns (79%) did attend the hearing screening and 242 newborns (21%) did not attend it, remaining with no explanation about their hearing condition. The data allow the conclusion that the nurse's performance scheduling the newborns for their newborn hearing screening at their discharge from the maternity, as well as their orientation accomplished at that moment, were important for the program's scope.

## LANGUAGE

### L001 Assesment of fluency in cluttering individuals

Bernardes, Ana Paula Lazarin^1^ – anapaulla_lb@hotmail.com

Broglio, Gabriela Aparecida Fabbri^1^

Capellini, Simone Aparecida^1^

Oliveira, Cristiane Moço Canhetti^1^

^1^ School of Philosophy and Sciences of Marília – UNESP.

This research is of extreme relevance in the area of fluency disorders, since studies involving cluttering are scarce. The objective of this study was to compare the fluency between fluent and cluttering individuals. The project was approved by the Committee of Ethics (N° 3491/2008). Ten individuals of both genders with ages ranging from 10 to 40 years participated until the moment, divided in two groups: GI - five cluttering individuals; GII - five fluent individuals. GI presented rapid and/or irregular speech rate and excessive typical disfluencies, whereas GII didn't present complaint of speech rate or disorders of fluency. It was used The Test of Fluency of the ABFW (Andrade, 2004), which characterizes the typology of the disruptions, the frequency of the disfluencies and speech rate. The partial results have shown that GI and GII presented the interjection (mean of 10.8 and 5 respectively) as the most frequent typical disfluency. The mean percentages of discontinuity of speech for GI and GII were respectively 14.2% and the 6.8%, and mean percentages of stuttering-like disfluencies (SLDs) were of 1.2% for GI and 0.8% for GII. The means of the flows of syllables spoken per minute had been of 316.5 for GI and 215.9 for GII, whereas the means of the flows of words spoken per minute were 174.7 for GI and 122.7 for GII. The results suggest that the difference between the groups for the disfluency is in the frequency of typical disfluencies, and not in the typology. The flow of syllables spoken as well the flow of words spoken per minute had been increased in the GI. The disfluencies can be caused due to the increase in the speech rate. This study represents a first attempt for the characterization of the profile of the fluency of clutterers. The objective measures used are important for the definition of the diagnosis, the treatment and the prognosis.

### L002 Communication skills in Noonan syndrome: case report

Costa, Erica das Graças^1^ – ericagrcosta@hotmail.com

Moya, Maria Paz^1^

Cusin, Dionísia Aparecida^1^

^1^Bauru School of Dentistry - University of São Paulo

The Noonan Syndrome (NS) is an autosomal dominant genetic disease characterized by dysmorphic changes with wide range of phenotypic expression and mental retardation of variable degrees. The objective is to verify communication skills of a female child, 3 years and 10 months old, with NS diagnosed by genetic examination. The family did not report neuropsychomotor or delay in language development. The child had not yet joined school. The following characteristics were observed: short stature, triangular face, ocular hypertelorism, anti-Mongoloid slope of the palpebral fissures, ear deployment low, dysmorphic craniofacial, neck and short winged. The evaluation consisted of the following: case history, observation of the communicative behavior, Peabody Picture Vocabulary Test (PPVT);Gesell and Amatruda's Developmental Scale –(GADS) and application of ABFW area of phonology and vocabulary. There was good understanding of specific and daily life contents. The patient was able to keep conversation, building affirmative, negative and interrogative sentences, making use of simple sentences for coordination and subordination, with shifts not always consistent and expansive. She demonstrated awareness of the verbal, nominal and gender agreement rules. She also made limited use of vocabulary and application of PPVT, failed to reach base. The GADS showed compatible results with her age and with the rough motor behavior. The fine motor, language, personal-social and adaptive behaviors were not compatible with her age, being less than expected. Phonological processes were not observed. There was difficulty with interpersonal interaction associated with episodes of temper tantrums. The communication skills are less developed than expected, with changes in semantic and pragmatic aspects. The difficulties of interpersonal interaction and social behavior may explain the limited change in the personal-social, adaptive and language levels reported. Considering the development of children with NS, it is expected an improvement of the social integration and learning with the beginning of the therapeutic process, instruction to the family and join of school.

### L003 Phonological disorders: case report

Zaac, Talita Bendasoli^1^ – talita.zaac@usp.br

Pereira, Cíntia Carolina^1^

Giglio, Lúcia Dantas^1^

Fukuda, Marisa Tomoe Hebihara^1^

^1^Ribeirão Preto School of Medicine – University of São Paulo.

There are difficulties in the utterance of speech sounds as the phonological disorder. This deviation begins during growth as part of the acquisition process and has unknown etiology. The objective is to describe the case of 3-year-and-4-month-old child with diagnosis of phonological disorders, treated at a Clinic-School of a Speech-language Pathology and Audiology course. The speech assessment was performed by observing the children in directed activities, in which behavioral, oral, listening and orofacial motor aspects were observed. The materials used in recreational activities were toys such as dolls, games, logical and temporal sequence, memory game, miniature pots and some food, puppets and illustrated children books. For assessment of phonology the ABFW test was used. It was observed that the cognitive, semantic, pragmatic, morphosyntatic were as expected for the age and the following communicative functions were also observed: regulatory, imaginative, personal, interpersonal, ideational. In the sample of spontaneous language there was a predominance of simple sentences, and complex sentences when they were more complex, the latter depending, at times, in an output expressed by the adult. Incomprehensible emissions occurred during the speech and in the analysis of the test ABFW there was predominance of the following phonological processes: placed in palatal, simplification of consonantal cluster, simplification of liquid and omissions. The other aspects of phonological assessment (orofacial motor and audiological factors) were found within the normal range. Based on the analysis of the results the child presented speech diagnosis of Phonological Disorder. We concluded that detailed speech evaluation allows diagnostic accuracy necessary for these cases and facilitates the development of appropriate therapeutic planning, facilitating the prognosis of the case.

### L004 Characterization of children with learning disorder and dyslexia through the Luria-Nebraska neuropsychology battery

Bretanha, Andreza Carolina^1^ – andrezabretanha@yahoo.com.br

Vieira, Millena Maria Ramalho Matta^1^

Crenitte, Patrícia Abreu Pinheiro^1^

^1^Bauru School of Dentistry – University of São Paulo.

It has been a growing complaint from the family, educational and clinical failure of children related to school learning to read and write, and some children are often wrongly named by parents and educators seeking justify their difficulties. The Speech-Language Pathology specialist is a professional qualified to understand and to dedicate to the treatment of these difficulties, but there is a shortage of materials to assist in diagnosis, especially materials that are intended for differentiation of the learning disorder and dyslexia. This study comes to take care of the clinical necessities, in view of the lack of reliable materials to help in the differential diagnosis of children with these disorders. Participants of this study were 30 children, 20 diagnosed with a learning disorder and 10 diagnosed with dyslexia at the age of 8 years to 10 years and 4 months, with no sensory changes, cognitive and behavioral. The Luria-Nebraska Neuropsychological Battery (LNNB-C) revised for children was applied, using only the scales: receptive language, expressive language, writing, reading, mnemonic processes, arithmetical dexterity and visual functions. The only function that has been made statistically significant comparison between the groups tested was the arithmetic skill, and that children with learning disorders had greater change that children with dyslexia. With regard to expressive language, receptive and memory, the change is made in proportion in both groups. In the reading function, the group with dyslexia showed greater change; the written and visual functions found a greater change in the group with a learning disorder. It was concluded that LNNB-C combined with other procedures is able to assist in the diagnosis of children with dyslexia and learning disorder and can be used as a tool for differential diagnosis for the reading, writing and mainly arithmetical functions, but other studies should are carried out for confirmation of the collected data.

### L005 Deaf inclusion and literacy: analysis of teaching practice

Schiavon, Daiane Natalia^1^ – daia_schiavon@yahoo.com.br

^1^University of State of São Paulo “Júlio de Mesquita Filho” – UNESP.

The educational inclusion requires a complete restructuration of the management actions and the actions of the entire educational system, which must ensure the necessary support to the conditions of each student, providing an appropriate educational response to individual needs. The research aimed to analyze the process of education of the deaf, period of literacy, observing the teaching of teachers and their communicative actions. This work was a research of qualitative nature only. An exploratory methodology was used, assisting in data analysis in order to formulate an hypothesis and improve the observations and ideas on the subject. Consisting of three Elementary Public Schools of a small-sizes city of the state of São Paulo, where three teacher/student dyads were analyzed in the first year of the elementary school. It was also applied a questionnaire/interview to teachers consisting of thoughts about their own pedagogical practice, regarding the teaching-learning of the deaf student. The results were analyzed and classified by specific categories concerning the teacher/student relationship, direct and indirect communication, activities, and interaction with the class. It indicates serious problems regarding the issue of establishing an effective channel of communication between the teacher and the deaf student; however, it also indicates that there is commitment and effort by both the student and the teacher so that this obstacle to the process of learning can be overcome. Such results reinforce the idea that it is up to the educators to be closer to the development of students, making adjustments to the curriculum and designing teaching communication strategies for each case. It is expected that the findings of the present investigation can serve as a tool for analysis and proposal for the development of specific teaching practices to the deaf student attending a regular class.

### L006 Performance of children from the 4th and 6th grades of elementary school in reading/writing and phonological awareness tasks

Koritiaki, Francine Dias^1^ – frandiko@hotmail.com

Santos, Patricia Leila dos^1^

^1^Ribeirão Preto School of Medicine – University of São Paulo

There are several studies reporting the importance of phonological awareness to the performance of reading and writing activities. What is not clear is whether this ability is a prerequisite for learning to read and write or if they develop it in interaction. To reach a conventional writing, and all the prerequisites necessary for literacy, it is necessary that the learner knows the spelling of the language aspects. Research on this subject has been developed with children in early literacy. However, some studies suggest that after completing the literacy, the development of metalinguistic skills continues, missing studies that investigate such continuity. The aim of this study is to compare the performance in reading, writing and phonological awareness among children of 4th and 6th grade of an elementary school. We applied the School Performance Test-TDE, Phonological Awareness Assessment Instrument for Sequential-CONFIAS and a standard text for dictation. The sample comprised 85 students, being 38 in the 4th grade and 47 of the 6th grade. The scores obtained in tests for 4th and 6th grade were, respectively, 21.3 and 28.3 in writing subtest of the TDE, 63.6 and 67.6 on the reading subtest, 52.6 and 56.6 in CONFIAS. As for spelling, the average amount of errors in the 4th grade was 37.9, while in 6th grade was 15.0. As expected, students in 6th grade had higher scores on tests TDE and trust, and lower incidence of spelling errors. However, it is worth highlighting that they have achieved low scores for what would be expected for the popular series. This research confirms that the greater the time of exposure to written language, the better the performance in the skills assessed and that the metalinguistic skills continue to develop even after the literacy.

### L007 Learning disorder as a result of epilepsy: case report

Moda, Isabela^1^ – isa_moda@yahoo.com.br

Kuroishi, Rita Cristina Sadako^1^

Mandrá, Patrícia Pupin^1^

Pacheco, Aline Cristina^1^

^1^Ribeirão Preto School of Medicine– University of São Paulo

Learning disorder refers to an internal dysfunction, in general neurological or neuropsychological, that manifests through specific difficulties in the acquisition and in the use of skills such as hearing, speech, reading, writing and mathematical logic reasoning. The Epilepsy fits within the disorder causal neurological factors and it is related to the changes of the oral and written languages. The objective of this study is to describe the case of a child, male, 83 years old, with speech pathologic diagnosis of learning disorder and medical diagnosis of epilepsy, consulted at the School-Clinic of a Speech-language Pathology and Audiology. It was performed on the child the evaluation of the cognitive aspects (problems resolution, initiative, reasoning and abstraction); communication aspects (functions of the oral language, phonetic-phonological, semantic, syntactic, morphologic, narrative and pragmatic); phonological conscience and the aspects of the written language (reading and production of graphemes). In relation to the cognitive aspects, the child presented reduced attention and concentration spam, slow reasoning, adequate capacity of problem resolution and symbolic game. In relation to the language's functions, the child presented communicative intention and used predominantly the expression function. In relation to the language formal aspects, the child presented phonological changes, and the semantic, syntactic, narrative and pragmatic aspects remained without alteration. In the written language, the patient presented alteration in the phonological conscience, in the fine motor coordination and low performance in reading and writing activities. Moreover, the patient presents history of behavioral disorder, low school performance with consequent abandonment and delay in learning. The findings of the Speech-language Pathology and Audiology evaluation are correlated with the characteristics of the learning disorder described in the literature. Therefore, it is concluded that the child evaluated in this study presents a learning disorder diagnosis concomitant with the neurological alteration. Based on this, it is relevant to point out the importance of the precocious speech pathologic intervention to prevent greater damages in the development of the oral and written language.

### L008 Knowledge of graphophonemic rules by students with and without learning difficulties

Fusco, Natália^1^ – nataliafusco@hotmail.com

Capellini, Simone Aparecida^1^

^1^School of Philosophy and Sciences of Marília – UNESP

Support: FAPESP

This study was for general objective to elaborate protocol of evaluation of reading based on the decoding of the rules of Brazilian Portuguese and for specific objectives to verify and to compare the level of knowledge of the students of 1st to 4th grade with and without learning difficulties how much with the use of the rules of the Brazilian Portuguese. 120 students of municipal public school participated, distributed of 1st to 4th grades, both genders, ranging from 7 to 10 years and 11 months of age, divided in 8 groups (GI to GVIII). As procedure the Protocol of Verification of the Level of Knowledge of the Rules of the Brazilian Portuguese was applied: Regular Word subtest (RW), Irregular Word subtest (IW), Incorrect Regular Words with Visual substitutions subtest (VW), Incorrect Regular words with phonological substitutions subtest (WP), Homophone Incorrect word subtest (WH) and Nonword subtest (NW). The results disclosed that all students of GI to GVIII presented statistically significant difference when comparing the expected and the actually received score, indicating that they had not gotten the maximum punctuation of rightness for these subtests. Regarding the comparison of the performance of the groups without and with learning difficulties, GI obtained better performance in relation to GV in the subtests of FW, RW, PW and VW. GIV had better performance than GVIII in relation to the subtests FW, IW and NW. Based on the obtained results it may be concluded that the tests were proven effective for verification of the level of orthographic knowledge of the students, demonstrating that students with learning difficulties presented flaws in the recognition of orthographic rules if comparison to students without difficulties.

### L009 Twin language: case study

Machado, Nathália Bócca Lourenço^1^ – nathaliablm_rn@yahoo.com.br

Oshima, Marluci^1^

Lopes-Herrera, Simone Aparecida^1^

^1^Bauru School of Dentistry – University of São Paulo.

There are several main approaches for the study of language development in twins. One of these approaches has been the investigation of twins that present language problems and display a phenomenon known as “secret language”. In the present research, a case study was made, being described the development of the language of two twin sisters in relation to secret language, to the phonological disorders that they had and its evolution during speech therapy. It was observed that in beginning of the therapy, the sisters had phonological omissions and substitutions: simplification of the archiphoneme {R} e {S} and omission the phoneme /r/. In addition to these alterations, one sister had omission and substitution of the phoneme /r/ and the other sister had omission of the consonantal group with /l/, being the only aspect that differentiated the language of both. With the evolution of the therapy, it was observed more development in one sister regarding the same phonemes worked in both, demonstrating that, prior to the therapy, the phonological systems were similar due to the narrow relationship of the pair of twins. This fact reduces the necessity of verbal development and diminishes the chances and motivation to communication, which led to the presence of the “secret language” between them and, from the beginning of the therapy, the environmental factor made possible the development of the individual characteristics in language development. From this study, it is reaffirmed that existence of genetic influence on the language of these children, but the environmental factors cannot be disregarded and must be considered the focus of the intervention.

### L010 Efficacy of phonological training program in students at risk for dyslexia

Fadini, Cíntia Cristina^1^ – cinfadini@gmail.com

Capellini, Simone Aparecida^1^

^1^School of Philosophy and Sciences of Marília – UNESP.

Support: CNPq

The development of receptive and expressive language of children in early literacy is a process that requires attention of educators, as the identification and early detection of signs of dyslexia and the problems arising from academic cognitive-linguistic changes can be minimized through the implementation of phonological training programs. The specific goal of this study was to verify the efficacy of the phonological training program in first-grade students prone to dyslexia. In this study we adapted the research about training phonological abilities developed by Schneider, Roth, Ennemoser (2000). The participants of this study were 30 students of the first grade, from both genders, ranging from 6 to 7 years and 11 months of age. All students were submitted to the test for early identification of reading problems; only 13 students showed difficulty in performing more than 50% of the test and were submitted to the training program. There were statistically significant differences, suggesting that 10 students submitted to the program showed better performance at a post-test when compared to pre-testing, showing, thus, that children at risk for dyslexia in this study actually had failures only in the literacy process, which justifies the use of programs like this to identify early problems in reading, reducing the number of unnecessary referrals to the completion of speech language diagnosis.

### L011 Children with complaints about school learning difficulties in a child neurology outpatient service: a possible relationship with the phonological processing abilities?

Furlan, Suzana Aparecida^1^ – suzi_af@yahoo.com.br

Kuroishi, Rita Cristina Sadako^1^

Fukuda, Marisa Tomoe Hebihara^1^

^1^Ribeirão Preto School of Medicine – Universiy of São Paulo.

The phonological processing abilities are characterized by the way information is processed, stored and used, and such abilities are necessary for the acquisition and development of school learning skills. Among the phonological processing abilities two are mainly highlighted: the phonological awareness which consists in the ability to discriminate and manipulate speech sounds; and the phonological working memory, known to mentally represent speech phonological characteristics in a short period of time. Thus, the present work aims at linking the performance of phonological processing abilities with learning abilities in children complaining about school learning difficulties. Ten 8-11-year-old children from 2nd to 5th grade of elementary school took part in this study: two of the subjects were girls and eight were boys. The children complained about school learning difficulties as they were cared in a neurology ambulatory of the Clinical Hospital of Ribeirão Preto Medical School/University of São Paulo (HCFMRP-USP). The following tests were used in the research: the Academic Performance Test (TDE), the Non-word Repetition Test and the Sequential Evaluation Instrument (CONFIAS). All the children underscored on the TDE. Regarding the phonological processing abilities, it was found that 50% of the sample were compatible to the pre-syllabic writing level of the CONFIAS and presented an average hit in five and six syllables lower than two hits in the Non-word Repetition Test. Both the phonological awareness and the phonological working memory are related with school writing, arithmetic and reading skills. In order to minimize de incidence of children with school learning difficulties, the professional responsible for the literacy of children should pay special attention to their phonological processing abilities.

### L012 Difficulties in the learning of the writing and the linguistic disorders of scholars in the city of Manaus-AM

Carvalho, José Luiz Brito de^1^ – jluizbrito25@hotmail.com

Korbes, Neodete^2^

Silva, Maysa Maura Feitosa da^2^

^1^ Bauru School of Dentistry – University of São Paulo.

^2^ Center University of North.

Phonological deviation is a linguistic disorder that half shows for the use of abnormal patterns in the language spoken. The objective of this work was to analyze the phonologic deviation during the learning of children from the 5^th^ year of the elementary education. The survey was accomplished in May of 2008 on 20 children of the Municipal Public School Léa Alencar located at municipal district of Manaus-Am. The children elaborated compositions through the thematic zoo figure presentation, which was retired from the book “Avaliação fonológica da criança: reeducação e terapia”, de *Yavas; Hernandorena; Lamprecht* (2002). The compositions were evaluated, and the 5 (25%) students who presented most orthographic errors were selected. The authors of these compositions were submitted to speech evaluation, by using the same thematic figure utilized to elaborate the compositions, and describing orally what they had seen in the figure. After that, the speech was analyzed by the phonetic transcription, and then the data were correlated with the orthographic errors and phonologic errors made during the speech. The results showed that: among the 20 children that made the composition, 15 (75%), presented orthographic errors, being 10 speller errors, 4 speller replacement and 1 speller addition; the speller most frequently unsettled were /r/ e /l/. Among the 5 (25%), children that participated to the second survey phase, with the speech recorded, 4 presented omission and 1 replacement. It was noticed that the most errors are related to the written when compared with speech, and all mistake made during the speech are present to the written. It was concluded that the difficult in the written, related to the significant errors during the composition, can be the casual factor the phonologic deviation.

### L013 Communicative skills in individuals with autistic spectrum deviations

Pedro, Aline Maria Aparecida^1^ – lypedro@yahoo.com.br

Santos, Lilian Maria dos^1^

Lamônica, Dionísia Aparecida Cusin^1^

^1^Bauru School of Dentistry – University of São Paulo.

The objective was to describe the communicative skills in individuals with aspects of autistic alterations. Twenty children from 3 to 13 years old, with autistic characteristics from the Speech Therapy Clinic of Bauru School of Dentistry, University of São Paulo participated in this study. It was approved by the CEP/FOB (042/2007). The instruments used were a Scale of Autistic Traits/Escala de Traços Autísticos (ETA) and Inventory of Communicative Skills/Roteiro de Habilidades Comunicativas (RHC). The data were collected based on the analysis of patients' handbooks. These data collection were based in indicators referring to the symptoms such as interaction aspects, social behavior and the incident of language alterations as for receptive and expressive processes. The statistical analysis was descriptive. In the ETA, the behaviors most out of step for the interaction were: maintenance of social exchange (100%); lack of the visual contact (90%), use of persons as instrument (90%), resistance to routine changes (90%), lacking of attention in productive activities (100%), difficulty to identify dangers (100%) and objects turn (70%). Poor adaptive behaviors that were observed: stereotypes (100%), physical balancing (75%), flapping (75%) and turn around itself (70%). In the RHC, as for the reception: difficulty for understanding linguistic contexts, even in concrete situations (100%). As for the expression: echolalia (50%), mutes (40%), jargon (35%); verbal stereotypes (70%); use of gestures for communication (5%). The characteristics involving behavior, language and social interaction are part of day to day activities, allowing this complex picture intervene with all communicative process. The communicative skills influences and are influenced by the presence of poor adaptive behaviors and interaction deficits. This casuistry presented serious alterations in the receptive and expressive processes of language with great impact in the activities of communication. The triad foreseen in these clinical pictures as for the communication alterations, interaction and behaviors makes this clinical spectrum a challenge and must promote reflections in search of therapeutic process that contributes to a better quality of life for these individuals.

### L014 The importance of the interdisciplinary approach in the diagnosis of the Stickler syndrome: case study

Stefanini, Marcela Rosolen^1^ – marcelastefanini@yahoo.com.br

Alvarenga, Kátia de Freitas^1^

Agostinho-Pesse, Raquel Sampaio^1^

Richieri-Costa, Antonio^2^

De-Vitto, Luciana Paula Maximino^1^

^1^Bauru School of Dentistry - University of São Paulo.

^2^Hospital for Rehabilitation of Craniofacial Anomalies – HRAC.

The association between the genetics and Speech Pathology is of utmost theoretical and clinical relevance. Thus, the characterization of the pathological case in complement to the genetic one aims at to the election of priorities of behavior and integrated forms of intervention. The present work consists of the presentation of a clinical case study in a clinical school of Speech Pathology. The child was attended in the clinic with two months of age for accomplishment of complete hearing evaluation, since she had failed in the test and re-test of the Transient Evoked Otoacustic Emissions. During the hearing accompaniment she was also evaluation of the language, with 8 months of age. The speech pathologist included the anamnesis and evaluation of the language focusing communicative comments of the behaviors and intentions in spontaneous situations, beyond the following scales: Gesell and Amatruda's Developmental Scale –(GADS) (GESELL, 2000) and Early Language Milestone Scale (ELMS) (COPLAN, 1993). The results showed in the speech pathologist's point of view, delay in the neuropsychomotor development and language. During the speech evaluation was also observed clinical signals like as horizontal nystagmus, buphtamos, dermatitis and infections of the recurrent respiratory ways, which suggest the hypothesis of an associated case the genetic syndrome. The child was sent to the geneticist, which confirmed the presence of a syndromic case, being diagnosed with the hypothesis of Stickler syndrome. The Stickler syndrome, focus of this study, is an autosomal dominant disorder, characterized by ophthalmological, articular, auditory and orofacial manifestations. It is caused by mutations in genes COL2A1, COL11A1 and COL11A2, responsible for the collagen synthesis. The gene spectrum has great phenotype diversity, which makes its diagnosis complex. Therefore, the use of a multiprofessional team for the diagnosis of these cases, including genetic counseling, is important.

### L015 Conceptions and attitudes of the teachers in relation to childhood stuttering

Salvini, Sandra Salenave^1^ – sandrasalvini@yahoo.com.br

Gonçalves, Marília Piazzi Seno^2^

Capellini, Simone Aparecida^1^

Oliveira, Cristiane Moço Canhetti^1^

^1^School of Philosophy and Sciences of Marília – UNESP.

^2^Center of Support psychology of the Municipal Secretary Education of Marília.

In Brazil, few studies have focused the question of the stuttering related with the school setting. The objective of this research is to analyze the knowledge of the teachers of the kindergarten public school have about stuttering and how they deal with this pathology, and; to elaborate and to distribute manuals of counseling on disfluency and stuttering to the participants of the research. The project was approved by the Ethics Committee (N°3496/2008). A total of 134 teachers of 19 schools of the kindergarten education, of whom 99.25% were female, took part in this research, with ages ranging from 21 to 55 years. A questionnaire and a brochure elaborated from the analysis of the answers of the questionnaires with counseling on disfluency and childhood stuttering were used. The result shown the stuttering was described as a speech pathology (79.10%) and emotional problem (51.85%). The causes of the pathology had been attributed to an emotional problem (62.23%), followed of anxiety (48.50%). The most commonly described characteristics of the stuttering were nervousness (45.90%) and shyness (44.43%). The majority of the participants (68.86%) believe that their attitudes can influence the stuttering of the child, for 94.81% the treatment of the stuttering is a speech-language pathologist job, and 97.65% believe that the stuttering has cure and it is prejudicial to school learning. Concerning the definition of the stuttering as a speech pathology and the belief in the cure of the disorder, the results had corroborated the study of Calais et al (2002). The answers had shown incoherence, therefore they had cited that the speech-language pathologist must carry through the treatment, however the pointed causes and manifestations say more respect to the emotional aspects. The results suggest that the teachers have many doubts on stuttering, and have interest in knowing the thematic better. We believe that from the offered counseling, the teachers will be able to propitiate an environment favorable to the development of the children who stutter in the school setting.

### L016 Risk factors for stuttering: comparison between children with and without family history of stuttering

Souza, Heloisa Aparecida^1^ – heloisarissato@yahoo.com.br

Cunha, Denise de Souza^1^

Santos, Ana Cláudia^1^

Giacheti, Célia Maria^1^

Oliveira, Cristiane Moço Canhetti^1^

^1^School of Philosophy and Sciences of Marília – UNESP.

The genetic aspect plays a significant role in the transmission of the susceptibility to stuttering, however, several are the factors that can act in a complex interaction and explain the origin of the disorder. The objective of this study is to compare the risk factors for stuttering in children who stutter with and without family history of stuttering. 60 children of both genders who stutter participated divided in two groups: GI - 30 children with family history of stuttering; GII - 30 children without family history of stuttering. The data were gathered through the Protocol of Risk for the Developmental Stuttering - PRGD (Andrade, 2006), which considers the following factors: age; gender; manner of onset and time of duration of the disfluencies; typology of the disfluencies; communicative and qualitative factors associates; physical and emotional stresses; family history of stuttering; personal, familiar and social reaction, and familiar attitudes. The data indicate that the groups differ from each other only in terms of communicative factors and emotional stress. GII presented a greater number of associated communicative factors than GI. In GI, 80% of the sample presented of emotional stress, compared with 90% in the GII. Another interesting findings was that the emotional stress presented by the GII had a higher score than the factors of the GI. The results corroborate the multifactorial aspect of the stuttering, that is, the transmission of this disorder seems to be related with some factors, and not only the genetic ones. However, children who stutter without family history of stuttering seem to demonstrate differences concerning quantitative and qualitative terms related to associated communicative factors and emotional stress. We can conclude that the results confirm the complexity of stuttering, as well as the necessity of investigating some risk factors for stuttering to understand the clinical case and to develop an adequate therapy.

### L017 Phonological awareness abilities in children with cleft lip and palate

Marcelino, Fabiana Carla^1^ – fabimarcelino@yahoo.com;

Feniman, Mariza Ribeiro^2^

Abramides, Dagma Venturini Marques^2^

Dutka, Jeniffer Cássio Rillo^1^

De-Vitto, Luciana Paula Maximino^2^

^1^Hospital for Rehabilitation of Craniofacial Anomalies – HRAC.

^2^Bauru School of Dentistry - University of São Paulo.

Researches indicate presence of speech production, language and understanding alterations in children with cleft. The ability to analyze the speech by phonological components is called phonological awareness and the phonemic analysis is the most complex. The phonological awareness development allows reading by the correlation between words and sounds. The aim of this study was to relate the language performance with phonological awareness in children with cleft lip and palate. The sample was composed of 24 children, from 7 to 9 years old, with complete unilateral cleft lip and palate, with palatoplasty accomplished until the 18 months of age, for the Furlow or Von Langenbeck surgical technique. The diagnostic process consisted of clinical and formal (Phonological Abilities Profile) speech and language pathology evaluation and cognitive (Raven's Colored Progressive Matrices) evaluation to discard possible associated alterations regarding general intelligence. In agreement with the accomplished speech and language pathology evaluation, it was observed that 18 children (75%) were diagnosed with language disorder, 9 (38%) children of those presented deficit in phonological awareness. Regarding the cognitive evaluation, only 2 children had results under the average. The results suggest a high percentage of alterations in oral language and phonological awareness, which may be related to the presence of cleft lip and palate on the studied sample. Such findings demonstrate the necessity of earlier evaluation and intervention in all aspects of oral and written language.

### L018 Phonological processes in a sample of students from Monte Negro/RO

Rodrigues, Raquel^1^ – queliita.rodrigues@gmail.com

Genaro, Katia Flores^1^

De-Vitto, Luciana Paula Maximino^1^

Merighi, Luciana Biral Mendes^1^

^1^Bauru School of Dentistry – University of São Paulo.

The phonological system is related to the speech sounds system and the rules to combine them in meaning unity. During this process, the child may produce phonological processes (PP) that affect a classes or a sequence of sounds. The aim of this study was to identify the PP occurrence and related them with age and gander. Three judges analyzed the sample of speech (imitation and picture naming test). The subjects were 83 students (44 female and 39 male) from Mato Grosso School, who lived in the urban zone of Monte Negro / RO, divided into two groups: GI, composed of 30 children, 6 to 7 years (15 girls and 15 boys), and GII, formed by 53 children between 8 and 11 years of age (29 girls and 24 boys). There was good agreement between the judges, ranging from 87% to 100% for the imitation and 88% to 100% for the picture naming test. The PP most frequently observed in the imitation test were: palatal fronting, fricative and stop devoicing, palatalization and velarization; while in the picture naming were: assimilation, palatal fronting, fricative and stop devoicing, palatalization, velarization, syllable deletion, liquid reduction, cluster reduction, final consonant deletion and gliding. There was a higher incidence of palatal fronting in males (picture naming) and GI (imitation). There was higher occurrence of gliding and final consonant deletion in GI (picture naming). So, there was a higher prevalence of phonological mistakes in boys and in young children. Most phonological processes found in the sample occurrence before the expected age.

### L019 Children reared in shelters: is it a risk for language development and hearing?

Franco, Elen Caroline^1^ – elen.fono@yahoo.com.br

Lopes, Andréa Cintra^1^

Lopes-Herrera, Simone Aparecida^1^

^1^Bauru School of Dentistry – University of São Paulo

In shelters, there is the absence of parents, but also the presence of caregivers that may have advantages for the development of children. The stimulation received by children is of paramount importance and also the environment in which the child is living in. The ability to communicate is essential for the emotional well-being and social adjustment, the full hearing is one of the conditions for the development of language. This research assessed the development of language of children who were in shelters and of who remained with the biological family. The verification of hearing was made by the investigation of possible risk factors for the language development. Participants were 30 children aged 14 to 47 months, 15 were living in shelters and 15 were children from public schools, all from a city nearby São Paulo. For assessment of language it was used the ADL Test (Assessment of the Development of Language) and, for the evaluation of hearing, it were used the pediatric audiometer PA5. Data analysis permitted to conclude that, among children in public school, 40% (n = 6) showed no language disturbance, 26.66% (n = 4) showed mild disorder, 13.33% (n = 2) moderate and 20% (n = 3) severe. Among children living in shelters, 26.66% (n = 4) showed no language disturbance, 20% (n = 3) showed mild disorder, 33.33% (n = 5) moderate and 20% (n = 3) severe. No child had problems identified by the hearing screening. It was concluded the disorders of language were more frequent in children sheltered, and this may be due to the situation that led to the institution, because the child is living a period of deprivation and, often, these places have no physical conditions and personnel to conduct activities that would promote the satisfactory development of these children.

### L020 Distance education: the use of a CD-ROM for teacher training

Oliveira, Ariádnes Nóbrega de^1^ – dine_usp@yahoo.com.br

Caldana, Magali de Lourdes^1^

^1^Bauru School of Dentistry – University of São Paulo.

The distance education has been a widely used tool for the dissemination of knowledge to more people. Whereas teachers are information multipliers and the most indicated for detecting early problems in speech class, this work has focused on teacher training using interactive CD-Rom on Communication Disorders. The aim is to produce a CD-Rom focusing on the aspects of language to be used with public teachers of Bauru-SP. In partnership with teachers from the public school system and a team of students of Computer Science, this CD-ROM was produced covering the development of children in school phase. The focus on content was the process of communication, speech and language, acquisition and development of language, changes in oral language, the importance of oral language for the development of written language and the role of teacher in stimulation and changes in the identification of speech, these subjects divided into modules and sub-modules, so the teacher could have easier access to the information. Regarding the technical aspect, it was prioritized the interactivity of the CD-ROM, making it challenging to acquire new knowledge from texts, pictures, videos and sounds. The final content was reproduced and distributed to the participating teachers. We can conclude that the material prepared for distance education was provided to teachers and encouraging the public school system to apply new knowledge in their daily activities, optimizing the quality of learning for their students.

### L021 Group of living: latent potential for the improvement of speech, quality of life and health promotion: a report of experience

Marcandal, Gessyka Gomes^1^- gessyka@yahoo.com.br

Baraldi, Débora Cristina^1^

Soleman, Carla^1^

Machado, Maria Aparecida Miranda de Paula^2^

^1^Federal University of São Carlos- UFSCar.

^2^Bauru School of Dentistry – University of São Paulo.

Language is an essential expression that human beings use to reaffirm their own individuality. Through the language, we get in contact and acquire the human culture and knowledge accumulated in the course of their social history. According to the Ottawa Charter (1986), Health Promotion is “the process of empowerment of the community to work on the improvement of their quality of life and health, including increased the participation in controlling this process.” The commitment of the Speech-language Pathology and Audiology to the population health promotion and the development of language transcend the traditional boundaries of clinical speech, public health and education, and surpass the demands of language changes so as to involve as subjects of the development of language practice, all citizens in their relations of coexistence and social contexts. This study aims to report the experience of the Harmony Group, a group of living that, using the handicraft, presented as a promising space for therapeutic work in order to work discursive practices, health education and thus improve the quality of the life of users of Guanabara and Jockey Club Family Health Units located at the municipality of São Carlos. The Harmony Group was coordinated by the Speech Pathologist resident and presented substantial data for the potential development of language structuring and construction of processes discussion, with consequent improvement of quality of life, increase of popular participation, self care and extension of social network, and also approximated the users and the professionals working in the Family Health Unit.

### L022 Characterization of the profile of children with cleft lip and palate treated at the HRAC/USP

Picolini, Mirela Machado^1^ – mirelapicolini@yahoo.com.br

Domingues, Ana Beatriz Cardoso^2^

De-Vitto, Luciana Paula Maximino^1^

^1^Bauru School of Dentistry - University of São Paulo.

^2^Hospital for Rehabilitation of Craniofacial Anomalies – HRAC.

Cleft lip and palate are the most common craniofacial anomalies. Their prevalence was estimated in 0.19 per 1,000 live births in Brazil, and the largest incidence is in the southeast region. The data on prevalence and incidence are limited and diffused, because the fast demographic growth and extensive territorial dimension. The aim of this study was to characterize the profile of children with isolated cleft lip and palate treated in HRACUSP, residents in the city of Bauru and regions. As a inclusion standard, children should be aged between 7 and 14 years old, regularly enrolled in primary school and are dedicated to the treatments performed at HRAC. The retrospective analysis of 61 medical records was accomplished between March and July of 2006. The analysis of the data showed that 52% of the patients were male, with an average age of 10 years and 2 months and 84% lived in Bauru. As for the type of cleft, 54% presented cleft lip and palate, 21% cleft palate, 18% cleft lip and 7% cleft submucous. Considering the heredity, absence the genetic inheritance was verified in 67% of the cases. The registers in the HRAC, in 80% of the cases were accomplished before the 12 months of age. Regarding the socioeconomic stratification, 70% were in the low class higher. Regarding the education, 70% were enrolled in classes from 1^st^ to 4^th^ grade and 30% from 5^th^ to 8^th^ grade of the elementary school. Having the age of 7 as the ideal reference for enrollment of a student in the elementary school, it was found that 82% of children did not present educational delay in years. The characterization of the population's profile was essential, since it enabled the search for possible factors associated with cleft lip and the development of educational and social.

### L023 Evaluation of the reading performance of 2nd to 6th grade students according to the complexity of the text

Dellisa, Paula Roberta Rocha^1^ – pauladellisa@hotmail.com

Navas, Ana Luiza Gomes Pinto^1^

^1^Faculty of Medical Sciences of Santa Casa de São Paulo – FCMSCSP.

The aim of this study was to describe the profile of reading fluency in children from 2^a^ to 6^a^ grades without learning complaints, according to the complexity of the texts. For this study 55 children were participants, submitted to the task of reading. Four texts were prepared, one consisting of short words, in other long words, a third with a simple syntactic structure and another of complex syntactic structure. Was asked to them to read texts, and evaluated various parameters of their fluency. Times' decrease was observed for the reading series, the text with short words presented short reading's time than the reading of long words, as the text syntactically simple for the complex. The variables “type of text” and “school” showed statistically significant differences as to the time of reading, with p = 0.011 and p = 0.023, respectively. As the rate of reading, there was significant increase in all the texts as the series progress, being higher in the text of short words in relation to the long words and syntactically simple in the complex. The percentage of words read correctly increased with education, as shown when compared between the initial and final series surveyed. The difference was statistically significant as the “education” in the four texts, with p = 0.011 and p = 0.001, respectively, for texts with short words and long, and syntactically simple and complex. As noted by Salles, Parente (2002), Macedo (2005) and Coelho (2008), there was decrease of reading's time in each grade. As with Capovilla et al (1998), the short words' text presented in all series less time than the reading of long words, showing the influence of the size of words in the time of reading. It is concluded that there was an effect on performance in reading according to the school, but this effect depended on the complexity of the text read.

### L024 Linguistic abilities in children with cleft lip and palate

Marcelino, Fabiana Carla^1^ – fabimarcelino@yahoo.com

Feniman, Mariza Ribeiro^2^

Abramides, Dagma Venturini Marques^2^

Monteiro, Camila Zotelli^1^

Dutka, Jeniffer Cássio Rillo^1^

De-Vitto, Luciana Paula Maximino^2^

^1^Hospital for Rehabilitation of Craniofacial Anomalies – HRAC.

^2^ Bauru School of Dentistry - University of São Paulo.

Researches indicate larger predisposition in children with cleft to present delay in the first words acquisition, in the short sentences production, difficulty in the words recovery, in the language understanding when compared with their pairs with normal development. The objective of this work was to evaluate linguistics abilities in children with cleft lip and palate. The sample was composed of 9 children (7 to 9 years old) with complete unilateral cleft lip and palate, with palatoplasty accomplished until the 18 months of age (Furlow or Von Langenbeck). The diagnosis process consisted of clinical and formal (school performance test, hearing processing and hearing attentions evaluation) speech and language pathology evaluation and cognitive evaluation (Raven's Colored Progressive Matrices). The data were analyzed qualitatively and quantitatively. In agreement with the accomplished speech and language evaluation it was observed 4 children with language disorder; observed alterations in the hearing abilities (attention, figure-grond, right and left selective attention, biaural integration, memory, speech secular process). The academic evaluation showed that 3 children presented inferior classification. This way, it may be concluded that children with complete unilateral cleft lip and palate presented oral language abilities inside of the normality, as well as, altered abilities, especially in phonology, semantics, syntax. Regarding the academic performance, most of the children presented appropriate abilities for the education. With base in these discoveries it is suggested the language evaluation in children with complete unilateral cleft lip and palate, to optimize the oral language and writing development.

### L025 Communication manifestations of an individual with Hypomelanosis of Ito

Silva, Aline Pillegi da^1^ – apsfono@gmail.com

Lamônica, Dionísia Aparecida Cusin^2^

Alvarenga, Kátia de Freitas^2^

^1^Program Graduate Special Edcation - Federal University of São Carlos.

^2^Bauru School of Dentistry – University of São Paulo.

Hypomelanosis of Ito (HI) is rare neurocutaneous syndrome characterized by spots in the skin, resulting from an alteration in the myelination, which can be associated with neurological, muscle-skeletal and ophthalmologic manifestations, and heart, verbal, genital and urologic abnormalities that can compromise the individual's development and maturation. The objective of this study is to report the verbal communication manifestations in a six-year and 3-month-old male child born from parents with no HI diagnosis, who presented with complaint of difficulty to understand the speech and communicate. The following procedures were carried out for the speech-language pathology and audiology evaluation: observation of the communicative behavior, Peabody Picture Vocabulary Test (PPVT), (Dunn & Dunn, 1981); ABFW - Test of Infantile Language (Andrade et al, 2000) and audiological evaluation by means of PEATE. The HI diagnosis was confirmed by phenotype and genetic examinations. It was observed that he presents limited time attention, superficial handling to objects and toys. He doesn't accept simple orders, he doesn't request adult's partnership, nor gives functionality to the objects, cries as protest. He doesn't look at who is speaking, nor demonstrates to understand when the stimulation is not concrete and the action is not immediate. In the TVIP he got the descriptive inferior decrease category. The following phonoaudiological processes had been found: fricatives plosivation, palatal frontalization, liquid simplification, final consonant and consonantal joint, deafness of plosives and fricatives. The audiological evaluation by means of eletro-acustics and eletro-physiological tests indicated normal functionality of the auditory system peripheral structures until encephalic trunk, with eletro-physiological threshold in bilateral 20dBNA. The literature on the subject is scarce with regard to the communicative abilities analysis and the development of these citizens deserves a careful analysis, seen the impact of the alterations in the communicative performance of these individuals with consequences in the activities of daily life, school levels and learnings.

### L026 The contribution of orofacial motricity to facial aesthetics

Sitta, Érica Ibelli^1^ – kek32@hotmail.com

Arakawa, Aline Megumi^1^

Oliveira, Ariádnes Nóbrega de^1^

Bassi, Ana Karolina Zampronio^1^

Caldana, Magali de Lourdes^1^

Miranda, Renata Puccinelli de^2^

^1^Bauru School of Dentistry – University of São Paulo.

^2^University Center of Araraquara – UNIARA.

The demand for treatment in facial esthetics has become a constant in the professional offices. Patients of both genders, concerned about a harmonious and rejuvenated face, goes to the orofacial motricity therapy clinics looking for this type of treatment. This study aimed to highlight the benefits of orofacial motricity therapy in a case study to provide aesthetic improvement in the patient's face with the performance of orofacial movement. It ran in a private clinic, in the city of Araraquara, after orthodontic intervention. This paper describes the analysis of the speech records of a female patient, 56 years-old, through anamnesis, initial assessment with phoptographs of the face, treatment, guidelines and final evaluation with photos for comparison. The data showed changes in orofacial muscles such as hypotonia, and inadequate function of mastication by preferential chewing on the left side and pain in the temporomandibular joint (TMJ) with dental pressure. The patient were subjected to 15 therapy sessions, including specific approaches of orofacial movement and mobility, guidelines for care in cleaning and protecting the skin, quality of daily food and the importance of sleep and well being. After knowing their inappropriate habits of facial expressions, therapies were worked eith the aim of providing muscle harmonization with relaxing and wrinkle smoothing massages. Once resolved the discomfort in the face by removal of the tooth clenching habit with the minimization of the points of tension and both articular and muscle pain, as well as changes in the masticatory pattern and quality of life, there was a consequent improvement in the harmony of both hemifaces. It is concluded that the benefits of the appropriateness of the orofacial muscles and stomatognathic functions together provide the balance of the morphology and function and consequently rejuvenation.

### L027 Interdisciplinary approach involving implantology and speech therapy: a proposal of performance, seeking the quality of life of patients with implant–supported prostheses

Silva, Andressa Sharllene Carneiro da^1^ – asc@usp.br

Santiago-Jr, Joel Ferreira^2^

Berretin-Felix, Giédre^1^

Pellizzer, Eduardo Piza^2^

^1^Bauru School of Dentistry – University of São Paulo.

^2^Araçatuba School of Dentistry – UNESP.

Implantology has as objective the implantation of alloplastic materials destined to support prosthesis in the jaws, aiming the rehabilitation of the patients in the context not only functional and aesthetic, but also to promote the social integration of the same. One of the specialist's requirements is the interdisciplinary integration and multiprofessional in the area of the health prioritizing a better quality of the patient's life. In this regard, the integration of Speech-language Pathology and Audiology and Dentistry in the treatment of the orofacial complex allows the normalization of the neuromusculature as a whole providing harmony of form and function of the stomatognathic system, offering stability and success to the patient's treatment. However, it was observed that there are still few references in the literature to approaches combining the specialties of Implantology (Dentistry) and Orofacial Motricity (Speech-language Pathology and Audiology), especially in edentulous patient. These studies are of great importance, since it has been observed the presence of oral myofunctional disturbances in patients rehabilitated with implant-supported prostheses, causing functional damages, mainly regarding the speech, mastication and deglutition. This way, the objective of this study was to accomplish a literature review in the Pubmed, ISI, Cochrane, Dentistry & Oral Sciences Source and Bireme databases having as inclusion criteria English- and Portuguese-Language studies published in the last five years. Two national and 14 international articles were found, approaching the need of integration of teams from both specialties in the patients' oral rehabilitation. It was concluded that the integration between Speech-language Pathology and Audiology and Implantology is necessary as a support therapy in extensive prostheses, in order to define goals and conducts in the rehabilitation of completely edentulous patients. An interdisciplinary approach should be part of the treatment plan for the placement of implant-supported prostheses, and other studies that add to the current literature should be accomplished lin order to evaluate cases in which a combined performance is necessary.

### L028 Clinical and videofluroscopic evaluation of swallowing in Guillain-Barré syndrome post-acute phase: qualitative and quantitative results

Ribeiro, Priscila Watson^1^ – priwtr@yahoo.com.br

Cola, Paula Cristina^1^

Gatto, Ana Rita^1^

Spadotto, André Augusto^1^

Silva, Roberta Gonçalves da^2^

Schelp, Arthur Oscar^1^

^1^School of Medicine of Botucatu – UNESP.

^2^School of Philosophy and Sciences of Marília – UNESP.

Acute inflammatory demyelination polyneuropathy, also known as Guillain-Barré syndrome is characterized by distal and proximal weakness. In many cases the first recognizable symptom is dysphagia due to involvement of cranial nerve. To describe the clinical and videdofluorscopic analysis of deglutition at the inicial recovery phase. The patient was a 25-year-old female with Guillain–Barré syndrome confirmed by electromyography and clinical neurological diagnosis. The patient had a nasogastric catheter and was traqueostomyzed. The clinical evaluation of swallowing was performed 54 days after diagnosis based upon the protocols proposed by Silva and Furkim (1999) and the blue dye test (Cameron et al, 1973), associated with videofluoroscopy evaluation of swallowing (Logemann JA, 1994). The degree of dysphagia was established according to Ott et al, 1996 and Daniels et al, 1997). The quantitative analysis of pharyngeal transit time (PTT) and oral transit time (OTT) were obtained using a specific software (Spadotto et al, 2008). It was observed depressed amplitude and strength of orophacial movements, reduced laryngeal elevation and no contrast leaking through the traqueostomy. There was also absence of signs suggestive of laryngtraqueal aspiration and/or penetration. In the videofluoroscopy, it was observed the presence of posterior oral escape, reduction of pharyngeal response, piriform recesses and valecule residues. No detection of laryngotraqueal aspiration and/or penetration. The average OTT was 5 seconds and the PTT, 3 seconds, both altered in comparison to normal parameters (Logemann, 1983). The presence of oropharyngeal dysphagia in this patient with Guillain-Barré syndrome suggests the possibility of initializing oral ingestion in earlier phase of recovering time. The treatment definition in these cases may be related to the severity of symptoms. Further studies must be done to determine the swallowing biomechanics during different treatment protocols.

### L029 Effects of orthognathic surgery on mandibular sensitivity and mobility

Passos, Dannyelle Christinny Bezerra de Oliveira Freitas^1^ – dannypassos@usp.br

Silva, Marcela Maria Alves^2^

Zanferrari, Elyria Oshiro^1^

Berretin-Felix, Giédre^1^

^1^Bauru School of Dentistry – University of São Paulo.

^2^São Carlos School of Engineering - University of São Paulo.

The treatment of dentofacial deformities involves the orthodontic treatment and orthognathic surgery and may cause changes in orofacial myofunctional conditions. The objective was to evaluate the mandibular sensitivity and movements before, 3 and 6 months after orthognathic surgery. The participants were 16 adults of both sexes with malocclusion and different facial characteristics, submitted to orthognathic surgery. In the evaluation of sensitivity, was used esthesiometry on cheeks, lips, tongue, mind, hard palate, soft palate and gingiva. The opening of the mouth, protrusion, lateral to the left and right were measured with a caliper. The results correspond to the preoperative evaluation of 16 patients and postoperative (3 and 6 months) of 7. Changes were found regarding the sensitivity of the gingival, hard palate and soft palate in the postoperative period of 3 and 6 months. About mandibular movements, when compared the pre-operative measures and 3 months after surgery measures, there was a decrease on the mouth opening (pre: x = 42.00 mm ± 7.90 mm after 3 months: x = 35.21 mm ± 8, 65mm), right laterality (pre: x = 5.43mm ± 1.27mm; post: x = 4.86 mm ± 2.27 mm), left laterality (pre: x = 6.43 ± 2.30 mm after 3 months: x = 6.07 mm ± 2.49 mm) and protrusion (pre: x = 4.86 ± 1 mm, 57mm, 3 months after: x = 3.00 mm ± 3.11 mm) but without statistical significance (p> 0.05). Comparing postoperative data of 3 (x = 35.21 mm ± 8.65 mm) and 6 months (x = 39.43 mm ± 7.74 mm) there was statistically significant increase in mouth opening (p = 0.02). The orthognathic surgery caused changes related to orofacial sensitivity and mandibular movements. spontaneous recovery did not occur in all cases in the follow-up period of this study.

### L030 Measurement of the pharyngeal transit time by a software in individuals after stroke

Cola, Paula Cristina^1^ – paccola@hotmail.com

Gatto, Ana Rita^1^

Silva, Roberta Gonçalves da^2^

Spadotto, André Augusto^1^

Schelp, Arthur Oscar^1^

Henry, Maria Aparecida Coelho de Arruda^1^

^1^School of Medicine of Botucatu – UNESP.

^2^ School of Philosophy and Sciences of Marília – UNESP.

Studies have demonstrated 50% to 76% incidence of oropharyngeal dysphagia in stroke. However, a few of these researchers have analyzed quantitatively the bolus displacement time among the phases of swallowing in these population. The focus of this study is to analyze pharyngeal swallowing transit time after hemispheric ischemic stroke. Forty nine right-handers post-stroke individuals participated in this study, 26 males and 23 females, aged from 45 to 81 years (mean age: 64 years). The ictus ranged from 1 to 30 days (mean of 6 days). The pharyngeal swallowing transit time was analyzed by digitalized videofluoroscopic images and measured by specific software. The measurement parameters of the pharyngeal phase proposed by Kendall *et al.,* in 2002, were adopted. The results showed that the pharyngeal swallowing transit time in stroke presented alteration, with mean of 2.8 milliseconds. There is a significant change in the pharyngeal transit time in stroke. Further studies are needed to assess the impact of this change in safe swallowing.

### L031 Anatomy and physiology of term newborn's suction and swallowing

Rondon, Silmara^1^ – silmara.rondon@usp.br

Berretin-Felix, Giédre^2^

Rodrigues, Antonio de Castro^2^

Daré Junior, Sergio^1^

^1^School of Medicine of University of São Paulo.

^2^Bauru School of Dentistry - University of São Paulo.

Knowledge of structural and functional characteristics of the stomatognathic system in newborns related to the process of breastfeeding is very important for health professionals. In this sense, was developed in the Discipline of Telemedicine of University of São Paulo an interdisciplinary project focused on training of professionals and health workers on breastfeeding, using technological resources to interactive education, which resulted in the need for the description of the anatomy and physiology of sucking and swallowing. Thus, the purpose of this study was to review the anatomical and physiological characteristics of the stomatognathic system of newborns to obtain theoretical basis for the development of digital instructional material and 3D iconographies. The bibliographic searches were conducted in scientific journals indexed in the LILACS and MEDLINE databases in the last five years, using the keywords: sucking, swallowing, breastfeeding, infant and anatomy. Textbooks of anatomy, physiology, Speech-language Pathology and Audiology and pediatric dentistry were also consulted. From the literature review were retrieved 13 scientific papers related, having been used the texts that contain information relevant to the construction of the roadmap: four papers and five book chapters. The text drawn up from the benchmark theoretical analysis consisted of anatomical descriptions on the baby's mouth, covering also the mechanisms that result in variations engine intraoral pressure during suction, and finally, the oral phase of swallowing. The review of the literature showed the scarcity of information geared to the anatomical aspects of the newborn's stomatognathic system, and that the outlined guidelines can direct not only the construction of dynamic three-dimensional images of breastfeeding in the virtual baby (www.projetohomemvirtual. com.br), but also result in the production of a text addressing issues that are not deeply discussed in the literature.

### L032 Oculo-auriculo-vertebral spectrum (Goldenhar syndrome): frequency and types of oral clefts observed in a sample of 34 patients

Goetze, Thayse Bienert^1^ – thatabg@yahoo.com.br

Zen, Paulo Ricardo Gazzola^1^

Rosa, Rafael Fabiano Machado^1^

Dall'Agnol, Lisiane^1^

Graziadio, Carla^1^

Paskulin, Giorgio Adriano^1^

^1^University of Health Sciences of Porto Alegre, RS – UFCSPA.

Oculo-auriculo-vertebral spectrum (OAVS - Goldenhar Syndrome) is a heterogeneous and variable condition, characterized by abnormalities involving the first branchial arches. Thus, alterations of face, as oral clefts, are common in the syndrome. The aim of our study was to determine the frequency and types of oral clefts observed in a sample of patients with OAVS, correlating this finding with the other clinical features presented by the patients. Our sample was composed by 34 patients, 22 males and 12 females, aged between 1 day and 17 years. All had normal karyotype and at least two features involving oral-cranial-facial, ocular, auricular and vertebral regions. A clinical data collection from their medical records was performed. Fisher exact test (P <0.05) was used for comparison of the frequencies. Oral clefts were verified in 12 patients (35%): 5 cases of cleft palate (2 of soft and hard palate, 1 of soft palate and 2 of submucous), 6 of cleft lip and palate (3 with bilateral cleft lip, 2 with right cleft lip and 1 with left cleft lip, all involving hard and soft palate) and 1 of longitudinal bilateral cleft lip. There were no statistically significant differences between the frequencies of the clinical features observed in the groups with and without oral clefts. The frequency of oral clefts in our study was similar to the majority of the works described in the literature that found rates ranging from 16 to 40%. Differences in this frequency seem to be related to the origin of the patients and the selection criteria used in studies. The great variability of the oral clefts observed called our attention, ranging from a bifid uvula to major cleft lip/palate. This finding, added to the other abnormalities observed in OAVS, justifies the participation of the speech therapist in the evaluation of these individuals, not only for the detection of the oral clefts, but also for their appropriate clinical management.

### L033 Immediate effects of the Pro-Fono® facial exerciser in patients with temporomandibular disorders (TMD)

Bisson, Mariela Heck^1^ – marielabisson@yahoo.com.br

Ferreira, Cláudia Lúcia Pimenta^1^

Felício, Cláudia Maria de^1^

^1^Riberão Preto School of Medicine – University of São Paulo.

Knowing the effects of the therapeutic procedures is a goal for orofacial myology area development. The aim of this study was to analyze the immediate effects of the Pro-Fono® facial exerciser in patients with temporomandibular disorders (TMD). The project was approved by the local Ethics Committee. Participated 11 patients with articular and/or myogenic TMD (EG, mean age= 34±14 years), randomly selected from a group of 20 patients and 10 control subjects (CG, mean age= 22± 3), matched by gender. All patients were examined for the classification according to Research Diagnostic Criteria for TMD (RDC/TMD) and surface electromyography (EMG) of the masseter and temporalis muscles in the diagnostic phase (PD). The instrument was the Freely, De Götzen. The EMG indices computed were muscular symmetry contraction (POC) and the standard total activity (IMPACT). The GE, after instructions, performed exercises as recommended in the package leaflet of the Pro-Fono® facial exerciser EMG was performed again (experimental immediate phase-PEI). The Student t-test was used for comparisons intra (paired data) and inter groups (non-paired data). The CG values were POCT= 88.43±1.16, POCM= 87.03±1.56 e IMPACTO= 107.09±22.4. In the EG, PD and PEI, the values were, respectively: POCT= 77.01±19.50, 78.35±11.48; POCM= 72.14±25.45, 69.59±25.05 and IMPACTO= 131.36±55.23, 99.55±34.23. There were no statistically significant inter-group intra-group differences (p> 0.05). The CG presented low asymmetry between right and left muscle pairs (POCM e POCT), and also the IMPACT value was compatible with that observed for control subjects. In the GE, the values were similar to those verified previously for groups with TMD and outside the limits of normality. There was considerable decrease in the IMPACT value of GE in the PEI, reaching the normality. The Pro-Fono® facial exerciser produced no significant immediate effect in EMG indices of the TMD group. The long-term of this device needs to be investigated.

### L034 Physiotherapeutic rehabilitation of mandibular trismus caused by odontogenic abscess

Vitoriano, Tamires Garcia^1^ – tamires_665@hotmail.com

Roldi, Armelindo^2^

Puppin, André Alberto Câmara^2^

Pinheiro, Tiago Novaes^1^

^1^Higher Education Unit Ingá – Uningá.

^2^Federal University of Espírito Santo- UFES.

The mandibular trismus is a very debilitating clinical event for the affected patient, usually associated with difficulty in opening the mouth, which impair speech and feeding. Its cause is multifactorial, related to traumatic events, infectious processes and temporomandibular joint disorder. The present study reports a case of mandibular trismus caused by an odontogenic abscess. The therapeutic measures involved the drainage of the abscess, rehabilitation with physiotherapy in order to gain mouth opening and endodontic treatment. Physiotherapeutic measures related to the gain of mandibular movement are discussed based on the pathogenesis of the lesion. The use of electrothermalphototherapy and prescription of muscle relaxants to treat the initial phase of muscle spasm are indicated. The treatment of clinical repercussions of mandibular trismus is multidisciplinary and involves dental, speech therapy and physical therapy approaches.

### L035 Effect of intravelar veloplasty on velopharyngeal function: perceptual and instrumental assessment

Silva, Andressa Sharllene Carneiro da^1^ – asc@usp.br

Gonçalves, Talita Fernanda^1^

Fukushiro, Ana Paula Fukushiro^1,2^

Yamashita, Renata Paciello^1^

^1^Hospital for Rehabilitation of Craniofacial Anomalies, University of São Paulo - HRAC-USP.

^2^Bauru School of Dentistry - University of São Paulo.

Financial Support: PIC-USP/RUSP.

Intravelar veloplasty (IV) is a surgical procedure used to correct residual velopharyngeal insufficiency (VPI), and its aim is retropositioning the velar muscles in order to promote adequate velopharyngeal closure. The objective of the present study was to verify the effect of IV on velopharyngeal function. Forty patients with repaired cleft palate±lip and residual VPI were evaluated, by perceptual and aerodynamic (pressure-flow technique) speech assessment before and after surgery. At the perceptual speech assessment the velopharyngeal function was classified as adequate, borderline or inadequate, based on hypernasality, nasal air emission and compensatory articulation scores. The aerodynamic assessment was performed in order to measure the velopharyngeal orifice area (VA) and determine velopharyngeal closure, classified as adequate, adequate/borderline, borderline/inadequate or inadequate. The statistical significance of differences between pre and post surgery values was verified by means of Wilcoxon test. Prior to surgery, all patients showed inadequate velopharyngeal function at the perceptual assessment, and, 92% presented inadequate velopharyngeal closure, at the aerodynamic assessment. After surgery, it was verified an improvement of velopharyngeal function in 48% of patients at the perceptual assessment which was confirmed in 35% by instrumental assessment. The difference between pre and post surgical values was statistically significant difference (p<0.01), in both perceptual and instrumental assessment. Thus, the IV has been proven an effective surgical procedure in improving IVF speech symptoms, yet in a non expressive proportion of patients. These findings strengthen the concept that the patients can be benefited from IV even when the resolution of the symptoms is not fulfilled. The palate muscle repositioning improves the velar movement reducing the symptoms as well as enhancing of speech intelligibility and favoring posterior treatments.

### L036 Speech therapy before and after orthognathic surgery for treatment of skeletal class II malocclusion and temporomandibular disorder

Moraes, Carla Fonseca^1^ — carla_fomoraes@hotmail.com

Koritiaki, Francine Dias^1^

Grechi, Tais Helena^1^

Mello-Filho, Francisco Veríssimo de^1^

Trawitzki, Luciana Vitaliano Voi^1^

^1^Riberão Preto School of Medicine - University of São Paulo.

The orthognathic surgery can be a treatment for temporomandibular disorder. This is possible in cases of dentofacial deformity because the skeletal repositioning promotes stimulation to the functional matrix for rearranging the muscle-skeletal relation and stimulating the remodeling. The speech therapy rehabilitation, in these cases, consists of favoring orofacial and cervical the functions, aiming at to a steady muscular balance. The objective of this study was to report the evolution of the orofacial myofunctional aspects in a case of dentofacial deformity and temporomandibular disorders. It is about a female patient, 31 years old, treated at the specialized service of a university hospital. The patient was submitted to the sagittal osteotomy of the mandibular ramus (mandibular advance) for correction of skeletal class II malocclusion and temporomandibular disorder. In this treatment, the evaluation and the speech therapy intervention were done pre and postoperatively. The treatment comprehended six months of therapy and the patient was instructed to do the exercises at home three times a day. The therapeutic planning was elaborated aiming at reducing the edema, relaxing the cervical musculature, orbicular upper/lower and mentalis, increasing of the tong muscular tension, improving of sensitivity and mandibular mobility. In the end of the rehabilitation it was observed that edema was absent, the more adequate hypoesthesia, orofacial musculature still present and bigger amplitude of the mandibular movements, therefore the measures with caliper evolved of 22 mm to 43.5 mm of spontaneous mouth opening, 4.5 mm to 6.5 mm of right laterality, 6.8 mm for 8.5 mm of left laterality and 4.5 to 7 mm of protrusion. The automated orofacial myofunctional characteristics in the patients with dentofacial deformities must be eliminated to guarantee the success of the result achieved through the orthognathic surgery. It was possible in this case to verify a favorable evolution in the orofacial myofunctional aspects.

### L037 Temporomandibular joint ankylosis post facial trauma: oral myofunctional aspects in speech rehabilitation

Almeida, Juliana Silva^1^ – jusial_2@hotmail.com

Vieira, Marília Diniz^1^

Grechi, Tais Helena^1^

Mello-Filho, Francisco Veríssimo de^1^

Trawitzki, Luciana Vitaliano Voi^1^

^1^Riberão Preto School of Medicine - University of São Paulo.

Ankylosis is defined as joint surfaces fusion. In cases of temporomandibular joint ankylosis with muscular rigidity among the joint surfaces, the mandibular motion is limited. Severity is related with the fracture type, time of the injury and the possibilities of growth in the period without treatment. The etiology is mainly related to direct or indirect trauma on temporomandibular joint, and references to the infectious and inflammatory or articular cases have also been found. The intervention over oral myofunctional therapy has as the main goal the direction of jaw mobility, muscle stretching, stimulation of the facial muscles and functioning of the stomatognathic system. The aim of this study was to describe speech rehabilitation with the interdisciplinary team, in a case of facial trauma that evolved to temporomandibular joint ankylosis. This is a retrospective study, using survey data from medical records, assessment of speech rehabilitation and oral myofunctional therapy. A 26-years-old female patient suffered a facial trauma due to traffic accident with multiple fractures involving the middle facial third and jaw. The injury evolved to bilateral temporomandibular joint ankylosis as diagnosed by the team of head and neck surgery at a university hospital. After surgery of arthroplasty of condyle and placement of silicone mesh surrounding the neck of the condyle bilaterally, the patient was sent back to speech rehabilitation. The speech rehabilitation procedures followed protocols with history, assessment and examination of face injuries. As a treatment plan in rehabilitation it was emphasized the reduction of postoperative swelling, pain relief, greater range of mandibular movements and recovery of oral functions within the possibilities of the case. Improvement was noted in the aspects emphasized, showing that the speech rehabilitation is necessary in cases of temporomandibular joint ankylosis combined with an interdisciplinary approach.

### L038 Follow-up protocol in oral myofunctional therapy of speech rehabilitation in a case of orthognathic surgery – Le fort I

Brandão, Bárbara Carolina^1^ – babicbrandao@yahoo.com.br

Freitas, Gabriele Nadal^1^

Silva, Janaína Bueno^1^

Grechi, Tais Helena^1^

Mello-Filho, Francisco Veríssimo de^1^

Trawitzki, Luciana Vitaliano Voi^1^

^1^Riberão Preto School of Medicine - University of São Paulo.

In adults, surgical-orthodontic correction in cases of dentofacial deformities seeks to restore harmony between jaw and maxilla. However, orthognathic surgery takes a changes in the structure balance of the facial skeleton, which can result in changes in the proprioceptive feedback mechanisms, especially the periodontal structures, changing the muscle activity. The functions of the stomatognathic system have to adapt the new forms, but they do not always occur spontaneously, and need specific therapy. The study describes the follow up protocol in oral myofunctional therapy in speech rehabilitation in a case of orthognathic surgery for maxillary expansion. The patient was a 17-year-old female with Class III dentofacial deformity. The pre-surgery evaluation of speech rehabilitation was made to investigate morphologic and oral myofunctional aspects and was found that the patient had indication of myofunctional therapy on this period. The orthognathic surgery Le Fort I osteotomy for maxillary expansion was performed in a university hospital by a interdisciplinary time. After the surgery, the patient was monitored in the bed and was guided by the speech therapist that informed about diet, and explained the utensils and consistency of food and how to reduce facial swelling. In each appointment, the patient was submitted to clinical evaluation and therapy that emphasizes reducing facial swelling on orofacial muscles and how to recover stomatognathic system functions. The patient also was submitted to the objectives examinations: bite force and maximum isometric tongue forces, before surgery, six months and one year after surgery. After 1 year of speech rehabilitation follow up, we noted improvement in oral myofunctional aspects checked by the objective and clinical orofacial evaluations.

### L039 Identification of the impairment degree of oropharyngeal dysphagia and the level of oral intake after stroke (CVA)

Bakr, Aline Ahmad^1^ – li.bakr@gmail.com

Cola, Paula Cristina^1^

Hordane, Gisele^1^

Carvalho, Lídia Raquel de^1^

Silva, Roberta Gonçalves da^1^

^1^School of Philosophy and Sciences of Marília – UNESP.

The cerebral vascular accident (CVA) is characterized as one of the most important causes of death and sequelae in our country, and the degree of impairment of oropharyngeal dysphagia (Daniels et al, 1997; Silva 1997 and 2004) as well as the level of oral intake (Crary et al, 2005, Silva et al, 2006; Furkim et al, 2008, Silva et al, 2009) have been previously studied. The purpose of this study was to identify and compare the degree of impairment of oropharyngeal dysphagia and the level of oral intake of individuals with stroke impairments in the diagnostic process. A retrospective analysis of 29 protocols for clinical evaluation of oropharyngeal swallowing of individuals who were evaluated in the oropharyngeal dysphagia clinic in a public service was conducted. Of these, 19 were male and 10 female, aged from 42 to 81 years; 24 had hemispheric strokes and 5 had brainstem strokes. The degree of impairment of oropharyngeal dysphagia was proposed by Silva (2004) and the oral intake scale proposed by Crary et al (2005). The statistical analysis was performed using the Goodmann test. It was found that the level of oral intake <5 (from exclusive alternative feeding to oral ingestion of two consistencies), the individuals had predominantly severe (75%) and moderate (25%) dysphagia (p<0.05). The level of oral intake> 5 (only oral intake) the dysphagia were predominantly moderate (53.3%) and mild (33.3%), whereas there were no severe dysphagia (0%) (p<0.05). During the oropharyngeal swallowing diagnostic process of individuals post-stroke with oropharyngeal dysphagia, it was observed inadequate guidance on the possibility of oral ingestion compared to the degree of dysphagia found in this study. This finding suggests the need for investigation in early oropharyngeal dysphagia post stroke, bedside evaluation, at the hospital.

### L040 Dysphagia and quality of life in individuals with stroke: a literature review

Brito, Gabriella Arioli^1^ – gabriella.brito@usp.br

Berretin-Felix, Giédre^1^

^1^Bauru School of Dentistry - University of São Paulo.

Neurological diseases, as stroke, can affect the orofacial functions, as well as the quality of life, because they cause interruption in one or more stages of the complex neuromuscular system connected to the several functions made by individual. The purpose of this work is to present a literature review about swallowing disturbances resulted by the stroke and its relationship with the quality of life. For such purposes, a search was made in LILACS, SciELO and MEDLINE databases in the last ten years, using the key words: “quality of life”, “stroke” and “swallowing”. The use of these three key words allowed finding of 15 international papers in MEDLINE database, but none of them was directly related with the subject of the research. The search in LILACS and SciELO databases did not result in any scientific article. Using “quality of life” and “stroke” it was found 43 national and 9 international articles. In the same way, researching “quality of life” and “swallowing” it was obtained 10 Portuguese-language papers and 4 English–language papers. Finally, the association between “stroke” and “swallowing” retrieved 22 national and 298 international articles. This results show that quality of life in stroke individuals is damaged. In dysphagic individuals, the quality of life has been studied specially in oncologic cases, patients with alteration in vocal cord mobility and elderly. Regarding swallowing, patients with stroke have oropharyngeal dysphagia, whose severity may result in aspiration, malnutrition and risk of death. In conclusion, it was not found researches in the literature associating quality of life, swallowing and stroke. It is important to develop works with this purpose since feeding problems have an important impact on people's quality of life.

### L041 Orthognathic surgery: myofunctional therapy in a post-surgical case

Yoshida, Fabio Shigueru^1^ – fabio.yoshida@usp.br

Ascencio, Ana Carolina Soares^1^

Berretin-Felix, Giédre^1^

^1^Bauru School of Dentistry - University of São Paulo.

Orthognathic surgery is indicated by oral and maxillofacial surgeons for the correction of maxillomandibular discrepancies and consists of the repositioning of the tooth bone bases. This surgery demands a series of adaptations for the patient due the changes in the oral morphology and frequent typical alterations of this procedure. In this way, the integrated treatment with the speech therapist specialized in orofacial myofunction occurs in order to normalize the functions, guaranteeing stability of the treatment and preventing possible relapses. The objective of this work is to present a clinical case of a 52-year-old female patient treated at the Speech Therapy Clinic of Bauru School of Dentistry, University of São Paulo. This patient was previously submitted to the procedures of mandibular advancement as well as advancement and superior positioning of the maxilla by the oral and maxillofacial surgeon. The clinical evaluation was carried out three months after the orthognathic surgery and evidenced orofacial myofunction disturbance characterized by the alteration of muscle contraction/relaxation status, alteration of mimic and face expression, atypical deglutition, chewing dysfunction, joint alteration and also mixed temporomandibular dysfunction and postural disturbance. Eight sessions were carried out for the whole treatment that included lip function, language, cheeks and mental muscles, increase of the mandibular mobility muscles, in addition to mouth opening and closing exercises and mandibular movements. A process related to awareness of the functions of chewing, deglutition and speaking was als carried out. The therapy results demonstrated remission of the temporomandibular dysfunction presented by the patient, improvement of mandibular mobility and development of the perception related to the orofacial functions. In such a way, the importance of the Speech-language Pathology and Audiology in cases of patients submitted to orthognathic surgery is largely proved, In the present case, the adjustment of the orofacial functions was necessary.

### L042 Perception and vocal damage in the voice of university teachers as a reality on the worker's health

Alves, Liliana Amorim^1^ – liliana@eerp.usp.br

Robazzi, Maria Lúcia do Carmo Cruz^1^

Ricz, Lilian Neto Aguiar^2^

^1^Ribeirão Preto School of Nursing – University of São Paulo.

^2^ Riberão Preto School of Medicine – University of São Paulo.

The perception of the vocal use might help the teacher to prevent possible vocal problems at work. The objective is to identify the signs, symptoms of the vocal changes presented by the teachers and strategies that they need to use in order to decrease these changes. This study was a quantitative research, comparing 58 teachers of a University in the state of São Paulo state. It was also used a questionnaire with data related to the signs, vocal symptoms as well as strategies that are necessary. Regarding the voice use at work, it was analyzed five hours maximum and 1 hour minimum per day, an outside work the time was 10 hours and the minimum as 1 hour. The voices were classified as adapted and deviated by the Speech Pathology. The main signs and vocal symptoms showed on the adapted voices were: 44.4% feel always tired/ and show a strong effort and always (FS), 55.6% show dryness FS, 77.78% present tight, pain and also burning never and rarely (NR). Regarding the deviated voices the main symptoms of voice changes were: 53.06% NR felt tired/effort 69.39% FS showed dryness; 59.18% tight NR, 79.59% pain NR and 87.76% burning NR. About the strategies and care with the voice, it was very clear that on the subjects with adapted voices that: 77.8% FS control the volume, 100% NR use amplificadores sonoros, 88,89% NR establish signs in order to ask for silence in the classroom, and 66.7% NR drink water in the classroom. As much as 79.59% of the ones with deviated voices presented FS voice control volume and 95.92% NR used microphones, but did not drink water. Teachers who participated on other studies also showed voice changes like in this study. There is a predominance of voice changes in university teachers and this change is directly associated with the presence of voice alteration detected by the Speech Pathology. Legend: (NR) never/rarely (FS) often/always.

### L043 Actors: communication skills in daily life and on stage

Baravieira, Paula Belini^1^ – paula_belini@yahoo.com.br

Arakawa, Aline Megumi^1^

Teles, Lídia Cristina da Silva^1^

^1^Bauru School of Dentistry – University of São Paulo.

The interpersonal communication skill has never been so necessary at the human relationship: professional, social and with families. In our society, not only individuals who domain certain techniques or knowledge stand out, but also those who are able to communicate in a clear and expressive way. the theater is an excellent ability communication laboratory, because by representing different characters we can live the expressiveness of communication in an intensive way. Do the theater actors have more skills in oral expression to their social communication? This study aimed to verify the actors' communication skills out of the stage and most commonly used intonation parameters in scene. 25 actors of both sexes, mean age of 22y4m±3years participated in the study. Fifteen of them are professionals of the theater group “Ruído Rosa”(USP) and “Quase Nove” (UNICAMP), having of 7y7m±2y5m of acting experience on average. Ten of them are amateurs with 6months±2m of acting experience on average in the theater group “Atuando em Psi” (students of the Institute of Physics of São Carlos - USP). Data were collected by a questionnaire containing 15 objective questions. It was observed that out of the stage the communicative behavior more often before entering the theater was the timidity marked by 61% actors, while 72% of them reported to be more communicative in their daily life after having enrolled in the theater. The social communication out of the stage becomes more expressive for 72% of actors and more creative and uninhibited to 61%of then. To prepare the character's expression, 80%of the actors use the intuition, 72% ask for the director's help and 4% ask for the speech pathologist helps. The vocal resources most reported for the characters expressiveness were: vocal intensity increase (72%), use of pauses (42%), frequency and voice speed increase (36%), decreased the use of voice speed and gestures (32%).

### L044 Comparison of acoustic measures before and after phonomicrosurgery of women with Reinke's edema

Reis, Nathália dos^1^ – fononana2@gmail.com

Ricz, Hilton Marcos Alves^1^

Aguiar-Ricz Neto, Lilían^1^

^1^Riberão Preto School of Medicine – University of São Paulo.

Reinke's Edema is laryngeal disease characterized by a chronic diffuse edema that extends the full length of one or both vocal folds, modifying the measures acoustics of voice. This study aims at comparative of measures acoustic of voice before and after first day of phonomicrosurgery of women with Reinke's edema. Before phonomicrosurgery, the participants were advised to stop smoking, received orientation of vocal and hygiene absolute voice rest. The acoustic measures of 6 30-45-year-old women, before and after the first day of phonomicrosurgery were seleted during the production of vowel /a/ in habitual frequency and intensity analyzed in the Multi-speech model 5105 computer program, from Kay Elemetrics®. In the studied sample, the mean fundamental frequency (F0) was 132.1 to 173.8 in the postoperative. The measures of PFR, Jitter (PPQ), vF0, Shimmer (APQ), NHR, DVB%, DSH% and DUV% increased in the postoperative course. After the phonomicrosurgery, the analysis of the F0 infers the reduction of the vibratory mass and the return of better conditions of vocal folds. The other parameter measures became worse, indicating the inability of the vocal folds to support phonation and to aperiodic vibration that can be found after surgery. It is concluded that after phonomicrosurgery that mean fundamental frequency returns the normal limits for sex and age.

### L045 Impact of the disfonia in the quality of life

Machado, Nathália Bócca Lourenço^1^ – nathaliablm_rn@yahoo.com.br

Brasolotto, Alcione Ghedini^1^

^1^Bauru School of Dentistry – University of São Paulo.

Quality of life is a concept related to the perception of the individual as to its position in the life in different contexts, being this subjective and abstract. Currently, instruments have been used to determine the global impact of the diseases and the treatments, considering the perspectives of the patient. The quality of life related to the voice demonstrates the real impact caused for these alterations in the life of the individuals and is important for the understanding of how the patient deals with these difficulties, in addition to providing information that could be used to offer for one better aiming of the treatment. It was objectified to investigate with a group of dysphonics individuals the data of the instruments validated in Brazil: Voice-Related Quality of Life (V-RQOL) and The Voice Handicap Index (VHI) to verify the specific contributions of these instruments in the practice of measuring the quality of life related to the voice. Data from 100 patients of the Speech therapy clinic of Bauru Dental School, University of São Paulo were analyzed and counting of the scores of both instruments was done in its different domains. The total score of the V-RQOL was 70% and for VHI was 37.6 points, standing out that the maximum punctuation is 100% and 120 points, respectively. The physical domain of the V-RQOL (62%) had higher score than the social-emotional (96%), and in VHI the physical aspect (18.5) scored more than the functional (8.63) and the emotional (10.3) aspects. The questions with highest score in the V-RQOL and the VHI were “I have trouble speaking loudly or being heard in noisy situations” and “My voice sounds creaky and dry” and “The sound of my voice varies throughout the day”. The protocols had indicated greater disadvantage in the physical/organic domain. But, the specific analysis of the questions sample that have different contributions and important contributions to be considered in the boarding with dysphonic patients.

### L046 Vocal and psychic changes in obsessive: compulsive disorder

Gurgel, Léia Gonçalves^1^ – leiagg@yahoo.com.br

Almada, Cecília Pereira^1^

Ferrão, Ygor Arzeno^2^

Cassol, Mauriceia^1^

Reppold, Caroline Tozzi^1^

^1^Federal University of Health Sciences of Porto Alegre – UFCSPA

^2^ Metodista University Center IPA

Phonoaudiological voice quality analysis involves auditory voice-perceptual and computed acoustic analysis. Obsessive-compulsive disorder (OCD) is a mental disorder included in DSM-IV as an anxiety disorder, showing population prevalence around 2.5%. The aim of the present study was to analyze psychological aspects and auditory and acoustic characteristics of the voice of patients with OCD. The sample consisted of 35 individuals, men and women, 17 with OCD and 18 without the diagnosis of OCD. The individuals were submitted to auditory voice-perception and acoustic analysis of the voice, as well as to a psychiatric evaluation that investigated depression and anxiety indicators. In the auditory voice-perception analysis, patients with OCD presented more frequent mild hoarseness of the voice, and resonance and pitch alterations. In the acoustic analysis, jitter was the only short-term disturbance measurement that presented significant statistical differences in this study. However, the shimmer value in patients with OCD has shown alterations. The results indicate that hoarse voice in individuals with OCD might be related to the use of antidepressant drugs, which have an antiobsessive effect, to the increase of muscular tension, to vocal abuse and emotional stress. Comparing the intensity of depressive and anxious symptoms through the average of the scores of Beck Scales for depression and anxiety, there was no significant differentiation between the OCD group and the control group. The results for shimmer (APQ) are related to the perceptual aspect of hoarseness. High pitch and rough vocal quality might indicate stress and tension. The data lead to the conclusion that a phonological and psychological intervention is promising for both diagnosis and treatment of the voice psychodynamic and self-esteem of these patients.

## Community health

### C001 Telehealth of the Bauru School of Dentistry: a descriptive analysis of the satisfaction with the course, according to the participants

Santos, Aline Robertina dos^1^ – alinesantos@usp.br

Silva, Andressa Sharllene Carneiro da^1^

Pinto, Ghiedree Fernanda Ramos^1^

Rizzante, Fabio Antonio^1^

Berretin-Felix, Giédre^1^

Blasca, Wanderléia Quinhoneiro^1^

^1^ Bauru School of Dentistry – University of São Paulo

In view of the professionals' heterogeneity in Speech Pathology and Audiology and Dentistry in different Brazilian territories, the Telehealth System of Bauru School of Dentistry tends to qualify technologies of information and communication that allows possible diagnosis, prevention and treatment of diseases, in addition to the education of the population and professional in health services. The aim of this work is to present the results of satisfaction of the students attending the Extension Course entitled “Telehealth System in Speech Pathology and Audiology and Dentistry” in 2008, approved as a Course of Diffusion by the Provost of Culture and Academic Extension of the University of São Paulo. It involves the undergraduates, graduates, professors and employees of Bauru School of Dentistry and the graduates and residents of the Hospital for Rehabilitation of Craniofacial Anomalies of the University of São Paulo. The selection of the 85 members was made through an exam, after an introductory course presentation. Activities were accomplished by class presence and at distance classes on different themes: basic computer science, construction of the scientific knowledge. The participants were stimulated to develop research projects and Teaching and Extension in Telehealth System. Forty-four members completed the course with monthly presence =85%. After 12 months the same members answered the satisfaction questionnaire about the activities developed. The satisfaction obtained was as follows: members' expectation (59%), course quality (97%), applicability (69%), workload (91%) and general objectives (75%). There was dissatisfaction in relation to the expectation (3%), applicability (6%) and reached objectives (2%). Other percentages referred to the partial satisfaction. Therefore, it was verified that there was high index of approval of the activities offered in 2008, except for the expectation level and applicability. It can be concluded that the objectives of the League are being reached in a gradual and satisfactory way, being important to identify and to solve the causes related to the withdrawing of some students.

### C002 Follow-up of hearing aid users in the Amazonian region

Arakawa, Aline Megumi ^1–^alinearakawa@usp.br

Sitta, Érica Ibelli^1^

Picolini, Mirela Machado^1^

Oliveira, Ariádnes Nóbrega de^1^

Bassi, Ana Karolina Zampronio^2^

Bastos, José Roberto de Magalhães^1^

Bastos, Roosevelt da Silva^1^

Blasca, Wanderléia Quinhoneiro^1^

Caldana, Magali de Lourdes^1^

^1^Bauru School of Dentistry – University of São Paulo

^2^ São Lucas University - RO

The Bauru School of Dentistry, University of São Paulo (FOB/USP) has developed since 2002 an university extension project in the city of Monte Negro (RO). The purpose of this work is centered in the speech, audiology and dentist service taken the health promotion and prevention in the population of this region. The hearing loss is considered a limiting problem when associated with communication and its impact can affect the well-being and people's relationships. With the development and use of individual sound amplification devices (hearing aids), the various effects caused by disabilities were minimized. Based on such aspects, the present study aimed to describe the quarterly follow-up in hearing aids users in the city and region of Monte Negro. For data collection, a monitoring questionnaire was developed addressing aspects related to the use of hearing aids, their current conditions and their ear mold as well as the social and economic conditions to access to batteries. This questionnaire was applied three months after the adaptation of the hearing aids. The data were analyzed subjectively according to the patients' report and the audiologist judgment. The results showed that 45.4% subjects reported using hearing aids throughout the day. Regarding the verification of the hearing aids and the ear mold conditions, it was observed that 95.5% of the equipments were functioning and that 45.4% of the ear molds were integrated and with flexible tube. Regarding the purchasing of batteries, 68.1% of the subjects reported difficulty in acquiring. As for the hearing aids and ear molds manipulation of hearing aids and ear molds, it was noted that 13.6% have difficulties in handling the hearing and 22.7% the mold. Considering these aspects, the patient's follow-up must be perfoemed by the FOB/USP team because the city does not have an audiologist as part of their Basic Health Center team.

### C003 Speech language pathology/audiology and dentistry experience in Rondônia

Oliveira, Ariádnes Nóbrega de^1^ – dine_usp@yahoo.com.br

Arakawa, Aline Megumi^1^

Xavier, Angela^1^

Bassi, Ana Karolina Zampronio^2^

Sitta, Érica Ibelli^1^

Rocha, Maurício Leonardo Margini^1^

Carvalho, Fábio Silva de^1^

Bastos, Roosevelt da Silva^1^

Bastos, José Roberto de Magalhães^1^

Caldana, Magali de Lourdes^1^

^1^Bauru School of Dentistry – University of São Paulo

^2^ São Lucas University - RO

In 2002, the project “USP in Rondônia” started its activities in the areas of Speech Language Pathology and Audiology and Dentistry in the city of Monte Negro, state of Rondônia, was found a lack of health care in both areas. Expeditions ran to this city to promote health, using a team of undergraduate and graduate students, professors and employees of the Bauru Dental School, University of São Paulo (FOB/USP). Before each expedition, students are prepared during three months with lessons about epidemiology, health policies, ethics, and the cultural and socioeconomic conditions of the local community. The aim is to improve the dental and speech therapy techniques of undergraduate and graduate students of FOB-USP in regions deprived of health care, and health promotion actions, with the aim of providing better health conditions for urban and rural people. The number of procedures and treatments in the dental and speech therapy was assessed. The procedures were performed individually at the Clinic of Oral Health and Speech Language Pathology and Audiology of ICB-5/USP. In rural area, it was done with the support of mobile dental clinic chairs and in partnership with the Family Health Program team. Since 2002, the number of speech therapy treatments has reached 5,319, which totalized a number of 16234 procedures. The dental patients were 7380 and the procedures, 19,552. The total number of patients was 12,699, and total of 35,786 procedures performed. Only in the year 2008, 3 expeditions occurred in this city. The speech therapy team treated 788 patients, performing 2,281 procedures. The dental team treated 1,798 patients, totalizing 3,603 procedures. The “USP em Rondônia” project, pursuit for health promotion, offering subsidies for the prevention and rehabilitation of dental and speech therapy diseases, therefore, improving the local population quality of life, which has few public health policies. Health promotion actions are important and still remain to be carried out to increase the benefits for this community.

### C004 Accessibility to products with xylitol in the prevention of otitis media in the city of Bauru, state of São Paulo

Xavier, Angela^1^ – angelax@usp.br

Arakawa, Aline Megumi^1^

Sitta, Érica Ibelli^1^

Carvalho, Fábio Silva de^1^

Bastos, José Roberto de Magalhães^1^

Caldana, Magali de Lourdes^1^

^1^Bauru School of Dentistry – University of São Paulo

Xylitol is a polyol used in oral hygiene products and chewing gum in substitution of sucrose because of its refreshing power and prevention of dental caries. It reduces the growth of *Streptococcus pneumonia*, a pathogen implicated in the etiology of otitis media and limits the adherence of *S. pneumoniae* and *Haemophilus influenzae* to cells of nasopharynge. The regular use of chewing gum with xylitol prevents otitis media in an extension from 30 to 42% in daily care with children. The mechanism of action of xylitol in the inhibition of growth of pneumococcus can simulate the mechanism of inhibition of growth of *S. mutans*. The present study aimed to evaluate the availability of oral hygiene products and chewing gum containing xylitol in its composition available in pharmacies and drugstores as well as large networks of supermarkets in the city of Bauru, state of São Paulo. Out of the 140 pharmacies and drugstores in the city of Bauru, state of São Paulo, 42 stores allowed the evaluation of oral hygiene products and chewing gum with xylitol, according to the regions of the city of Bauru. Moreover, large supermarkets were included to be evaluated, where 17 stores allowed the research. Products easily found with xylitol were children's toothpastes without fluoride, which were found with greater availability in the central and south region of the city. The supermarkets visited did not provide oral hygiene products with xylitol for the population. There is little variety and accessibility to oral hygiene products and chewing gums with xylitol for the population, but there is a growing tendency for the advertisement of these products in the market.

### C005 Instruments for quality evaluation of health websites: a preliminary study

Souza, Patrícia Jorge Soalheiro de^1^ – pattysoalheiro@hotmail.com

Bastos, Bárbara Guimarães^1^

Ferrari, Deborah Viviane^1^

^1^Bauru School of Dentistry – University of São Paulo.

The easy and fast access to health related information on the internet makes it an ally in the process of patient education. To achieve it, there must be guarantee that available information is reliable and relevant, therefore the need for evaluate the website quality. Many instruments do not have good usability, demanding a lot of time for its fulfillment and being difficult to understand. This study compared the usability of different instruments for health related website's evaluation. Five participants were asked to navigate the website “*Saúde na Internet*” and evaluate it through three different instruments administration: Emory (36 items), Michigan (43 items) and HonCode adapted to Portuguese (7 items). The time spent and results obtained by the application of each instrument were compared. The participants' comments and perceptions regarding instrument's use were qualitatively analyzed. The mean time for instrument's application were 2.2 (HonCode), 11 (Emory) and 13 minutes (Michigan). For all instruments, used it was observed variability of scores inter-participants, this being higher for the Michigan questionnaire. There was discrepancy in the results provided by different instruments – Emory and HonCode classified the website as being adequate, while the Michigan classified it as being “weak”. The website classification obtained by the instrument application not always agreed with subjective ratings of website quality given by the participant, especially for the HonCode. The Michigan questionnaire was considered difficult to understand and too time consuming. The Emory was considered the easiest to use and understand and provided the most reliable website classification. Combining the strongest features of different tools might be useful for both the development and evaluation of health information on the Internet.

### C006 Application of IOI-HA questionnaire after the fitting of hearing aids in Monte Negro-RO

Arakawa, Aline Megumi^1^ – alinearakawa@usp.br

Picolini, Mirela Machado^1^

Sitta, Érica Ibelli^1^

Oliveira, Ariádnes Nóbrega de^1^

Bassi, Ana Karolina Zampronio^1^

Caldana, Magali de Lourdes^1^

Bastos, José Roberto de Magalhães^1^

Blasca, Wanderléia Quinhoneiro^1^

^1^Bauru School of Dentistry – University of São Paulo

The Bauru Dental School, São Paulo University enrolled expeditions in the city of Monte Negro, state of Rondônia, for health promotion and prevention. The project is being developed since the 2002 and counts on the participation of undergraduate and graduate students and Speech Pathology and Audiology and Dentistry professors. Regarding the hearing heath, it is known that the success of fitting hearing aids involves many aspects, such as including improvement in speech recognition, users benefit and satisfaction. The purpose of this study was to carry out a subjective evaluation regarding to aspects of the hearing aids, after three months of fitting. Eighteen patients with hearing loss fitted with hearing aid in the Clinic of Oral Health and Speech Language Pathology and Audiology of Rondônia participated in this work. The subjective evaluation involved the application of the International Outcome Inventory for Hearing Aids (IOI-HA) - translated into Portuguese spoken in Brazil by Bevilacqua et al. (Cox et al., 2002). The questionnaire consists of seven questions, which assess the outcome of adaptation under the following: use, benefit, activity limitations, satisfaction, residual participation restrictions, impact on others and quality of life. From the sample's total, 38.9% uses the hearing aids more than 8 hours per day, 44.4% reports that helped considerably, 33.3% have little difficulty in carrying out activities, 66.7% thinks it very important using hearing aids, 33.3% reported that the hearing aids did not affect their activities, 55.5% reported that their hearing loss does not affect other people and 38.9% thinks that the hearing aids has changed their life. The subjective evaluation showed that the use of hearing aids has contributed to a better quality of life of these patients.

### C007 Ethical aspects in speech-language pathology and audiology course

Carleto, Natalia Gutierrez^1^ – natalia.carleto@usp.br

Arakawa, Aline Megumi^1^

Santos, Cibele Carméllo^1^

Bretanha, Andreza Carolina^1^

Sitta, Érica Ibelli^1^

Beraldinelle, Roberta^1^

Sales-Peres, Arsênio^1^

Sales-Peres, Silvia Helena de Carvalho^1^

Caldana, Magali de Lourdes^1^

^1^Bauru School of Dentistry – University of São Paulo

The bioethics teaching in health graduate courses is an issue that needs to be discussed, considering the scientific and technological development. The bioethics can be applied in Speech-Language Pathology and Audiology courses, which treat the problems related to communication that influences patient's quality of life. The aim of this study was to identify which Brazilian Speech-language Pathology and Audiology graduate course has the bioethics, ethics and/or deontology disciplines in their curriculum, and verify in which year of the course it is taught. Data were obtained from CFFa, online, and were accessed from March to April 2009. Twenty-two institutions were found at CFFa, related to the State of São Paulo, but 4 had no curriculum, 1 had website address problem and 3 had no bioethics, ethics and deontology in the curriculum. Those disciplines were distributed differently in each institution, over the program. The conclusion is that no institution adopts the three terms and there is no consensus on which period they must be given. More studies are needed about this theme and reflections about bioethics and Speech-language Pathology and Audiology are required.

### C008 Undergraduate students as research subjects: ethical and legal aspects

Sitta, Érica Ibelli^1^ – kek32@hotmail.com

Beraldinelle, Roberta^1^

Bretanha, Andreza Carolina^1^

Carleto, Natalia Gutierrez^1^

Arakawa, Aline Megumi^1^

Santos, Cibele Carméllo^1^

Sales-Peres, Arsênio^1^

Sales-Peres, Silvia Helena de Carvalho^1^

Caldana, Magali de Lourdes^1^

^1^Bauru School of Dentistry – University of São Paulo

It is indispensable to define the selection criteria of the subjects who compose a research sample because it influences directly in the qualitative aspects as well as in their confidentiality. Undergraduate students are usually the target of studies and are many times used without well established criteria. This review aims to discuss the condition of undergraduates as research subjects as well as the role of researchers based on the ethical and legal precepts established. In spite of the researchers having knowledge of ethical rules and following the legal aspects, they end up distancing themselves from the respect to autonomy of the research subjects as well as neglecting the defense of their vulnerability. The undergraduates, on the other hand, act in a passive manner, avoiding questioning. Therefore, these results clearly show the need for guidelines that protect this group of participants for the development of science in the universities.

### C009 Orientation to public schools teachers of the eastern area of Porto Alegre on vocal health issues

Etges, Camila Lucia^1^ – camila_etges@hotmail.com

Martinez, Chenia Caldeira^1^

Koehler, Cristine^1^

Reis, Mariana Citton Padilha dos^1^

Fávero, Samara Regina^1^

Goetze, Thayse Bienert^1^

Bortolini, Vaneila^1^

Marini, Ana Lucia Sant'Anna^1^

Cassol, Mauriceia^1^

^1^Speech Patology/Audiology School – Federal University of Health Sciences of Porto Alegre (UFCSPA).

The voice disorders, and its impact on teacher's health, affect the daily activities of teaching, which contributes to the decline in their quality of life. Thus, among the risk factors for voice problems, there are inadequate conditions of work, excessive working hours, lack of information about the correct use of voice and low demand for specialized care. We analyzed the complaints of public school teachers who, and were advised about vocal hygiene. The intervention consisted of guidelines on vocal health, and occurred from April to September of 2008. This was held in Health Fairs in public schools of the eastern area of Porto Alegre. The activity, organized by undergraduates from the Speech Language Pathology and Audiology course of the Federal University of Health Sciences of Porto Alegre (UFCSPA), was initiated by a screening of associated symptoms to the inadequate use of voice and followed by a dynamic group, which intended to guide the teachers on ways to handle the voice and prevent future vocal problems. At the end of the activities, an informative folder was distributed, and apples and glasses of water were offered to the participants in order to enhance their benefits to the voice. Statistical analysis was performed from the SPSS program version 13.0 and p-values by chi-square test and Pearson correlation. Thirty-four teachers with a mean age of 43 years were analyzed. A correlation was observed between vocal fatigue and vocal effort (p = 0.006); burning and discomfort in the throat, (p = 0.004); sore throat and hoarseness (p = 0.001). These results corroborate the literature, stating that the presence of these symptoms is characteristic of vocal abuse and teachers complaint which impairs the health and quality of life.

### C010 Knowledge of health professionals of units of community health care services of Conceição Hospital Group about the speech-language pathologist action

Reis, Mariana Citton Padilha dos^1^ – marianacpdosreis@gmail.com

Koehler, Cristine^1^

Keitel, Patrícia^1^

Bortolini, Vaneila^1^

Gadenz, Camila Dalbosco^1^

Bier, Bianca de Almeida^1^

Nery, Andresa Ramos^1^

Pinto, Maria Eugênia Bresolin^1^

Damiani, Lizandra Konflanz de Lima^1^

^1^Speech Patology/Audiology School – Federal University of Health Sciences of Porto Alegre (UFCSPA)

In Brazil, the precariousness of the public health system in care of patients with communication disorders occurs because of insufficient human resources and the restricted participation of Speech-Language Pathologist (SLP) in the implementation of public health programs. Besides, there is in the health care units a lack of knowledge about the SLP and its role. Consequently, this lack of information and the need of new intervention measures for an effectiveness of SLP contribution in interdisciplinary groups limit the participation of this professional in primary health care and in the health units. This study intends to analyze the knowledge of health professionals inserted in units of Conceição Hospital Group about the role of Speech and Hearing in public health. This research is a transversal study, in which the analyzed factor will be collected by structural interviews with 80 health professionals, inserted in 12 units of Community Health Care Services of Conceição Hospital Group, located in the northern area of Porto Alegre, Brazil. None of these units have the SLP service. After the interviews, information papers about the subject will be distributed. This study will be conducted by students of Speech-Language Pathologists, which integrate the Tutorial Education Program in Health (PET-Saúde, Programa de Ensino Tutorial em Saúde) of the Foundation Federal University of Health Sciences of Porto Alegre (UFCSPA). The collected data will be analyzed by the software SPSS version 12. It is expected that the results corroborate with similar studies previously conducted, which demonstrated a lack of knowledge of some specialties about Speech Language Pathology and Audiology and its possibilities of intervention in primary health care.

### C011 Speech language pathology and audiology therapy in a child educational center: report of experience

Pacheco, Aline Cristina^1^ – lili_e_vc@hotmail.com

Bisson, Mariela Heck^1^

Kuroishi, Rita Cristina Sadako^1^

Lupoli, Luciana da Mata^1^

Mandrá, Patrícia Pupin^1^

Moda, Isabela^1^

Pazetto, Lilliam Fernanda^1^

Picinato-Pirola, Melissa Nara de Carvalho^1^

Reis, Nathália dos^1^

Santos, Ariana Elite^1^

Trawitzki, Luciana Vitaliano Voi^1^

^1^ Riberão Preto School of Medicine – University of São Paulo

The nursery environment must be specially designed to offer the children in their first years of life, the ideal conditions that provide and encourage their development in an integrated and harmonious way. The speech pathology therapist's role is strategic for the communication development in children in a nursery, because he/she can act orienting educators and professionals about the communication aspects and about the communication development. The aim of this study was to report the speech pathologic therapy performance in public health focusing in optimizing the children oral communication. For three months, 20 children of a Child Educational Center (CEC) participated in workshops, who presented, in the screening speech performed, an oral language development less than expected. The workshops consisted of hearing training, optimization of oral language and stimulating exchanges of discussion and non discussion shifts. The mini-courses were conducted with all children of the CEC (except those in the nursery), through drama with the objective of showing the damage of vocal abuse and deleterious oral habits. After the mini-courses, it was performed the activity of the right and wrong, with the objective of strengthening the awareness performed on them. The participating children achieved the objectives proposed in the workshops, in others words, by optimizing the language they were able to internalize the strategies that have been worked. The teachers also reported to the trainees the progress of children, observed in the classroom. During the courses, the children remained attentive and outlined some communication attitudes regarding the performances. Positive results from the courses were seen during the activity of “correct and incorrect” because the children showed a large number of right answers in this activity. The experience has contributed positively to the learning and training of the working team. According to this experience, it was possible to understand the importance of the work of the speech pathology therapist directed to CECs regarding the optimization of communication.

### C012 Speech therapist formation as a matrix supporter for the multiprofessional residence: report of experience

Baraldi, Débora Cristina^1^ – debsbaraldi@gmail.com

Dressler, Carla Viviane Georg^1^

Marcandal, Gessyka Gomes^1^

Ribeiro, Alexandra Carlos^1^

Sgobin, Graciela^1^

Silva, Rubem Abraão^1^

Soleman, Carla^1^

Toso, Michele^2^

^1^Federal University of São Carlos- UFSCar.

^2^State Hospital of Ribeirão Preto.

The Residence, a postgraduate formation based on service training, has been developed with the support of state departments, municipal departments and the Ministry of Health, and aims to bring professional training in health and social reality of work in the basic health care unit, qualifying professionals to work in the system. The Multidisciplinary Residence Program in Family and Community Health (PRMSFC), of Federal University of São Carlos (UFSCar), in partnership with the Municipal Secretariat of Health of São Carlos, has the Speech Therapist as a professional who works under the logic of matrix support, which consists in a new strategy for the reorganization of work in health. This paper aims to report the experience of training the speech therapist as matrix supporter in the Family Health Strategy (FHS) at the PRMSFC of UFSCar. The insertion of the Speech Therapist in PRMSFC UFSCar is to in-service training, in a team with different types of actions, involving theoretical and practical activities that are conducted in multidisciplinary groups, or specific nucleus for discussion of “practices in health” and the reflection about the pratice. The operationalization of the matrix support has some obstacles already cited in the literature and experienced in this process. Therefore, the actions of the residents are developed in order to fit the local reality seeking to meet the needs of the network. In everyday practical of this residence program, it has been sought, through the logic of matrix support, building a training process and working with different ways. The role of the speech therapist in primary care, more precisely in the Family Health Strategy, under this logic, is the construction of a new speech praxis. Thus, the mode of residence in-service training, focusing on support matrix, represents a potential strategy for this rethinking of the training in health.

### C013 The living group as a strategy for improving interpersonal relationships in a family health team of São Carlos city: a report of experience

Marcandal, Gessyka Gomes^1^- gessyka@yahoo.com.br

Baraldi, Débora Cristina^1^

Soleman, Carla^1^

Machado, Maria Aparecida Miranda de Paula^2^

^1^ Federal University of São Carlos- UFSCar.

^2^Bauru School of Dentistry – University of São Paulo.

The aspect of integrality in health care emphasizes the importance of teamwork in the Family Health Strategy (FHS), and to make possible to this strategy triggers a process of construction of new practices it is essential that workers articulate a new dimension in the development of teamwork. This dimension requires the interactive relationship between workers, mediated by the exchange of knowledge and dialogue. The design and type of work can be presented in two forms: group or team. The configuration of a group is the first condition to exist, between people, a social interaction and some kind of link. The passage of the condition of group to a team is the transformation of “common interests” in “interests in common.” This study aims to report the Workshop of Living as a strategy to improve the interpersonal relationships of staff of Family Health of Guanabara and Jockey Club in São Carlos city. Considering the difficulties of interpersonal relationships between members of teams, was proposed by the Speech-language Pathology and Audiology resident the “Space of the Word,” a workshop of Living, which was the background to make handicrafts during the discussion of issues concerning the daily life of the subject, the teamwork and dialogue, and making possible, in addition, a space-sharing among professionals in the FHS. This process has provided changes in the relationship among members of the team, characterizing the passage of a condition of “cluster team” to “team groups” which reflects at the full care of their users, since focusing on collaboration between the health professionals, integrating efforts, stimulating discussion and exchange of information in the care of users and the proposals for improving the problems of the community for all professionals in the team.

### C014 Young doctor Bauru project

Correa, Camila de Castro^1^ – camilacorrea@usp.br

Martins, Aline^1^

Souza, Patrícia Jorge Soalheiro de^1^

Barros, Guilherme Toyogi Tanizaki^1^

Fernandes, Gabriela^1^

Sant'Ana, Nicolle Carvalho^1^

Ferraro, Gyovanna Junya Klinke^1^

Zabeu, Julia Speranza^1^

Pinto, Ghiedree Fernanda Ramos^1^

Silva, Andressa Sharllene Carneiro da^1^

Blasca, Wanderléia Quinhoneiro^1^

Berretin-Felix, Giédre^1^

Ferrari, Deborah Viviane^1^

De-Vitto, Luciana Paula Maximino^1^

Lamônica, Dionísia Aparecida Cusin^1^

Alvarenga, Kátia de Freitas^1^

Brasolotto, Alcione Ghedini^1^

Spinardi, Ana Carulina Pereira^1^

Meyer, Adriana Sampaio^2^

^1^Bauru School of Dentistry – University of São Paulo.

^2^Hospital Rehabilitation of Craniofacial Anomalies - University of São Paulo.

The Young Doctor Project acts in the valorization of the prevention through Interactive Tele-education. It uses resources of Telemedicine, education at distance and the “Man Virtual” Project in order to motivate elementary and high school students to do health promotion projects. The purpose of this project is to involve the students in prevention and intervention activities about hearing and vocal health, providing knowledge to the public. It was developed in October 2008 in the elementary school “Criarte” located in Bauru-SP. Seventeen 8th grade students of the elementary school participated by watching lectures and taking part in dynamic and interactive activities, organized by professors and students of the Course of Speech-Language Pathology and Audiology of Bauru Dental School and professionals of the Hospital for Rehabilitation of Craniofacial Anomalies of the University of São Paulo. Those students, the “Young Doctors”, applied the acquired knowledge for the classmates during a period of 5 days. In the last day, there was a Cultural Fair opened to the community. Materials such as the “Virtual Man” (3D graphic computation), panels, and embroidered jackets were used. Of the 150 people that visited the event, among parents, professors, students and others, 53 answered a questionnaire that verified the satisfaction level. Excellent satisfaction was reported by 66% of the visitors, while 28% classified the exhibition as very good, 0.6% as good and 0% found the exhibition weak or irregular. Therefore based on the visitors' reported interest and judgments provided by the “Young Doctors”, it may be concluded that the hierarchy knowledge can provide a better life quality for the population favoring the prevention of possible disturbances in communication.

### C015 Speech-language pathology and audiology in public health: the importance of the speech therapist in the health family program

Martins, Cristiane^1^ – chryspts@msn.com

^1^Faculdade Ingá (UNINGÁ) – Maringá, PR, Brazil

The work of the Speech-Language Pathology and Audiology in the Basic Health Care Units (BHCUs), especially in the Family Health Program (FHP) teams, is growing every day; however, there is still a lot to be conquered in terms of Public Health. With the objective of investigating possible speech-language pathology and audiology alterations assisted by the FHP, this tried to elucidate the functioning of Public Health System (SUS) and the FHP, their goals and the insertion of the speech therapist in this system. Some theoretical models and practical performance were addressed. This study was developed in the city of Nova Londrina at the homes of families assisted by the FHP program. For the accomplishment of this research it was analyzed the 30 protocols answered by the mothers and from children. From this total 15 were male and 15 were female, between the ages 6 to 10 years. The results identified the main Speech-Language Pathology and Audiology alterations: deleterious habits such as use of nursing bottle 76,67% (23 in 30), regular pacifier 70.00% (21 in 30); breathing alterations: 66.67% (20 in 30) presented nocturnal mouth breathing and 53.33% (16 in 30) presented diurnal mouth breathing; the difficulties is speech were 56.67% (17 in 30) of the subject and 43,33% (13 in 30) associated with ear inflammation/infection. It is know that the alterations found in 6-10-year-old age group can interfere in the alphabetization process and that if it is not prevented or treated early a delay in this process may occur. From the data obtained in this study it is possible to recognize the need for a speech therapist in the FHP teams in order to explain and advise the population about the alterations in communication.

### C016 Relationship between diadochokinesis, maximum phonation duration and fundamental frequency in adults

Beraldinelle, Roberta^1^ – roberaldinelle@yahoo.com.br

Modolo, Daniela Jovel^2^

Silvestre, Andriele Pereira

Berretin-Felix, Giédre^1^

Brasolotto, Alcione Ghedini^1^

^1^Bauru School of Dentistry – University of São Paulo.

^2^Hospital Rehabilitation of Craniofacial Anomalies - University of São Paulo.

The phonoarticulatory and respiratory functions are interrelated, and is important in clinical assessment of speech and voice, the integrated analysis of the tests that analyze them. This study aimed to relate the values of Maximum Phonation Duration (MPD) and Fundamental Frequency (f_0_) with the values of oral and vocal fold diadochokinesis (DDK) in 150 individuals, 20 to 49 years, divided into subgroups of 10 to 10 years. It was done the analysis of DDK (“a”, “i”, “pa”, “ta”, “ca” and “pataca”) of the MPD (“a”, “i”, “u”, “s”, “z” and the relationship s / z) and f_0_ (vowel “a” sustainable, counting numbers, and repetition of the vowel “a” and “i”). The results of the DDK and the MPD, as well as the DDK laryngeal and f_0_ were compared by Pearson correlation coefficient, whereas significance of 0.05. It was observed that how much higher was the MPD, more was the DDK (for men: “ta” x “numbers”, “a” x “u”, “a” x “z”, and “the” x “numbers” for women: “pa”x”s”, “pa”x”z”, “ta”x”z”, “ca”x”i”, “ca”x”u”, “pataca”x”s”, “pataca”x”z”, “a”x”z”, “i”x”s”, and for some emissions from groups divided by age and gender). For the 20 to 29 year olds, in the emissions “a”, “pataca” for man and “pa” for women, how much higher was the ratio s/z, lower was the DDK. About the relationship between DDK and f0 for men aged 30 to 39 years, the faster the recurrence of “a”, the more acute the frequency of speech, and the higher the DDK of “ i “, the higher the f_0_ of speech, sustained vowel and repetition of the vowel “i”. Some relationships between DDK and MPD were found. Although no systematic relationship was found between the DDK and f_0_, the study has relevant information concerning the trends presented in the tasks of DDK, which should be considered in clinical assessment and may be useful in the therapeutic process.

### C017 Vocal characteristics of men and women aged 50 years and older

Santos, Aline Oliveira^1^ – alineoliveira.s@hotmail.com)

Brasolotto, Alcione Ghedini^1^

^1^Bauru School of Dentistry, University of São Paulo

The studies about modifications due to aging have been contributing to the understanding of the vocal aspects in the old community. However, the period of appearance of the characteristics of the vocal aging in men and women is not clear. The purpose was to know the values of the acoustic parameters and to compare them among Brazilian Portuguese speakers of both genders aged 50 years and older, subdivided in decades. There were extracted values from average fundamental frequency (f_0_), standard derivation of (f_0_), jitter, shimmer and Noise Harmonic Ratio (NHR) during the emission of three vowels “a” supported per 3 seconds of 161 subjects: 70 men and 91 women with ages between 50 and 79 years. The emissions were recorded in studio and analyzed by the computer-based system Mult Dimension Voice Program (MDVP) model 5105, by Kay Elemetrics. Statistical analysis was done by two-way analysis of variance. There was no statistically significant difference between the ages for all parameters, for either men or women. However, it was possible to observe for the 50, 60 and 70-year-old men respectively, the gradual increase of jitter (0.81; 0.95 and 1.55%), shimmer (3.82; 4.32 and 5.13%) standard derivation of f_0_ (1.67; 2.27 and 3.12Hz), which did not happen to women. Only f_0_ and standard derivation of f_0_ (respectively 125,97Hz and 2,35Hz for men and 197.01Hz and 5.00Hz for women) presented statistically significant difference (p<0.000 and 0.013%), and thus for jitter, shimmer and NHR, there was no significant difference between the types. It was concluded that considering the division of decades like subgroups, there was no statistically significant difference as for the acoustic parameters analyzed for subject from 50 years. Further studies are suggested with wider age groups and vocal analysis procesudres in order to understand the development of the characteristics of vocal aging.

### C018 Hearing evaluation of young people who use portable digital music players

Santos, Izabella dos^1^ – (izabella.santos@terra.com.br)

Couto, Christiane Marques do^1^

^1^Faculdade de Fonoaudiologia - UNICAMP

The use of portable digital music players is common among young people. Normally this kind of equipment is used in high volume, which can damage the users' hearing. So, this research evaluated the hearing of young people who use these equipments and measured the sound pressure level which they are exposed to. During the research, young people were submitted to a pure-tone audiometry, including high-frequency, immittance measurement and microphone tube measurement. In the end, young people received guidance about their hearing health. 19 university students, with mean age of 22.21 years, participated in the research. They used their equipment 2.36 hours per day on average with a mean volume of 76.10% of the total, generally in noisy environments, like buses. The audiometry showed, in both ears, that hearing thresholds were higher on the frequencies of 6, 8, 12.5 and 16 KHz. Despite the high thresholds of 6 and 8 KHz, they were still within the normal range. However, for the high-frequency audiometry it is still a pre-established pattern. In impedance, 100% of young people showed type A on tympanometry. The ipsilateral and contralateral acoustic reflexes were found with an average differential over 90 dB. Measuring the sound intensity which young people are exposed to, it was observed that the level of sound intensity near the tympanic membrane is similar for both ears. For the right ear, a maximum value of 79.33 dBSPL (250 Hz) and a minimum value of 46,88 dBSPL (8000Hz) were found; for the left ear, a maximum value of 79.11 dBSPL (250 Hz) and a minimum value of 51,08 dBSPL (8000Hz) were the results. However, some cases have reached peaks of 98.2 dBSPL (1000 Hz). Therefore, it may be concluded that, even not having a hearing loss, these young people are exposed to a high sound pressure for a long period of time, which may become a hearing damage risk.

### C019 Oral and laryngeal diadochokinesis rehabilitation in elderly submitted to implant-supported rehabilitation in the mandibular arch

Pulga, Marina Jorge^1^ – marinajp@usp.br

Berretin-Felix, Giédre^1^

Brasolotto, Alcione Ghedini^1^

^1^Bauru School of Dentistry – University of São Paulo.

Aging allied with tooth loss and use of ill-fitted dentures can cause changes in orofacial functions, in particular the neuromuscular coordination during speech. This study used tests of oral and laryngeal diadochokinesis (DDK)before and after oral supported implant rehabilitation of 10 patients, been five women and five men with a median of 64 years, total edentulous and users of total removable prostheses. Were considered the vowels “a”, “i”, the syllable “pa” for analysis of parameters of the average rate of the DDK (mT), the standard deviation of the period of the DDK (dpP), the coefficient of variation of the period of the DDK (cvP), disturbances of the period of DDK (jiT) and coefficient of variation of intensity peak of the DDK (cvI), and finally, the trisyllable “pataka” analyzing the total number of emissions and the number of “pataka” per second. Statistical analysis was performed using the Student's t test for two paired groups. The laryngeal DDK showed no statistically significant variations, while the oral DDK showed decrease in the average time of vocalizations (p = 0.024), improve of the ability to maintain constant the intensity of vocalizations (p = 0.041), increase in the total number of emissions “pataka” (p = 0.003) and in the number of “pataka” per second (p = 0.010). Thus, we conclude that the oral rehabilitation by means of supported implant prostheses heightened oral neuromuscular coordination in elderly edentulous and users of removable complete dentures.

### C020 Assessment of phonological working memory: comparison of performance in different age groups

Grivol, Márcia Aparecida ^1^- marcia.grivol@usp.br

Hage, Simone Rocha de Vasconcellos ^1^

^1^Bauru School of Dentistry – University of São Paulo.

Working memory is a system of processing and storing information for short-term that keeps the thought, learning and communication, is necessary for the realization of complex cognitive activities such as comprehension, lexical access and learning of reading and writing. It is believed that the memory is expanded with age until they suffer a decline by the aging process, even if healthy. Studies involving adults and elderly normal allow early identification of possible losses in memory allowing early intervention and better conditions for individuals with impaired working memory. Whereas the skills of phonological working memory extending until certain age and can decline with aging, this study aimed to verify the performance of individuals at different ages, without changes of language in tasks that assess the phonological working memory (Test of Repeat Non Words). The study involved 90 normal people, 30 children (between six and eight years and 11 months), 30 adults (between 19 and 35 years) and 30 elderly (60 years or more). It was applied the Test of Nonword Repetition, which is to repeat 40 words invented from 2 to 5 syllables. The results were analyzed statistically with descriptive measures and paired t test, considering a significant p value <0.05. In the NonWord Repetition Test total score, there were statistically significant differences among the different age groups (elderly <children <adults). The conclusion was that elderly had worse performance when compared to adults and children, suggesting that phonological working memory suffers decline with the aging process. On the other hand, adults had better performance than children, showing that adults have better storage of verbal material compared to children.

### C021 Telepractice: hypermidia system development on phonological therapy for distance learnig

Spinardi, Ana Carulina Pereira^1^

De-Vitto, Luciana Paula Maximino^1^

^1^Bauru School of Dentistry – University of São Paulo

Speech Pathology, like other areas of health, is developing clinical and educational activities at a distance, trying to follow the current scientific and technological development. So, Telepractice (use of information and communication technologies in Speech Pathology) becomes an alternative to promote the integration and value of practice. Considering the irregular distribution of speech pathologists in the country and the concentration of educational institutions in specifics areas there is a need to develop Distance Learning (DL) programs to distribute the information of a regular mode giving priority to quality of life of communication disordered people. The instructive material is an invaluable tool in DL programs because it is establishing the educational relationship. Thus, its content must be presented in a dialogue and context form to promote meaningful learning. The aim of this study is present a hypermedia system develop to be used as a teaching resource to the phonological therapy teach. A “Therapeutic Procedures in Phonological Disorder” CDROM was produced by interdisciplinary work of speech pathologists and web designers and it was developed in 4 stages: analysis and planning, conceptual, navigation and interface modeling and implementation. It was used hypertext to respect style of learning of each student, a DL assumption. The student profile, definition of goals and various media integration (sound, video, image, and animation) were considered in developing a material. Using this system, students/speech pathologists will be able to acquire, store and use the information in practice. The CDROM validation will be made with Speech Pathology students from different universities.

### C022 Relationships between phonological process and reading and writing alterations in subjects with specific language impairment

Nicolielo, Ana Paola ^1^ – (anapaolanicolielo@yahoo.com.br)

Hage, Simone Rocha de Vasconcellos^1^

^1^Bauru School of Dentistry - University of São Paulo.

The phonological processing (PP) refers to the use of the phonological information in the processing of the language and it is composed for three abilities: phonological working memory; lexical access and phonological conscience. The occurrence of limitations in these abilities has been pointed as being a cause of phonological deficits in children with Specific Language Impairment (SLI), and for consequence, the alteration in the written language presented for these subjects. The objective of the study was to verify if exist relations between PP with reading and writing difficulties in the SLI as well as pointing witch ability of the PP more are related with such difficulties. They had participated of the study 20 subjects with SLI and 20 with Typical Language Development (TLD), with ages between 7 and 10 years, being submitted to the following procedures: reading and writing tests of the Writing and Reading Analysis Test (TALE), nonword repetition subtest of the Phonological Memory Test, Rapid Automated Naming (RAN), and the Profile of Phonological Skills. The statistical associations were carried out by the chi-square test. The results point that the subjects with SLI present significantly worse performance (p=0.05) in the PP abilities than the subjects with TLD. The statistical associations evidenced that no ability stood out in the relation, being that all presented strong association with the difficulties of reading and writing in the SLI. Therefore, the study evidenced that limitations in the PP are related with the difficulties in the written language, however, there was no outstanding ability in this relation. We suggest that long-term studies are performed with the objective of verifying whether the stimulation of the three abilities of the PP carried out in the preschool phase affect positively the stages of development of the written language in the SLI cases.

### C023 Anatomy and physiology of suction and swallow in term newborn

Rondon, Silmara^1^ – silmara.rondon@usp.br

Berretin-Felix, Giédre^2^;

Rodrigues, Antonio de Castro^2^;

Daré Junior, Sergio^1^;

^1^School of Medicine of University of São Paulo;

^2^Bauru School os Dentistry - University of São Paulo.

Knowledge of structural and functional characteristics of the stomatognathic system in newborn related to the process of breastfeeding is very important for health professionals. In this sense, was developed in the Discipline of Telemedicine of University of São Paulo an interdisciplinary project focused on training of professionals and health workers on breastfeeding, using technological resources to interactive education, which resulted in the need for the description of the anatomy and physiology of sucking and swallowing. Thus, the purpose of this study was to review the anatomical and physiological characteristics of the stomatognathic system of the newborn to obtain theoretical basis for the development of digital instructional material and 3D iconographies. The bibliographic searches were conducted in scientific journals indexed in the databases LILACS and MEDLINE, the last five years, using the keywords: sucking, swallowing, breastfeeding, infant and anatomy. Were also consulted textbooks of anatomy, physiology, speech therapy and pediatric dentistry. From the literature review were conducted 13 scientific papers related, having been used the texts that contain information relevant to the construction of the roadmap: four papers and five book chapters. The text drawn up from the benchmark theoretical analysis consisted of anatomical descriptions on the baby's mouth, covering also the mechanisms that result in variations engine intraoral pressure during suction, and finally, the oral phase of swallowing. Through the work of revision developed it was noted the scarcity of information geared to the anatomical aspects of the stomatognathic system in the newborn, and that the roadmap drawn can direct not only the construction of dynamic three-dimensional images of breastfeeding in the virtual baby (www.projetohomemvirtual. com.br), but also result in the production of a text addressing issues raised little in the literature.

### C024 Proposal of assessment protocol for candidates children at cochlear implant with cerebral palsy

Santos, Maria Jaquelini Dias dos^1^; majadisa@hotmail.com

Bevilacqua, Maria Cecília^2^;

Moret, Adriane Lima Mortari^1^;

Lamônica, Dionisia Aparecida Cusin^1^;

Yamaguti, Elisabete Honda^2^;

Costa, Orozimbo Alves^2^;

Vassoler, Trissia Maria Farah^2^;

^1^Faculdade de Odontologia de Bauru – USP; ^2^Centro de Pesquisas Audiológicas - Hospital de Reabilitação de Anomalias Craniofaciais – USP.

International studies relate that is large the number of children with hearing impairment and other associated impairments of development. We focus the cerebral palsy which is characterized as the most frequent motor disorder in early childhood, being the hearing impairment one of the associated disabilities. The main of this study is the aplication of the Proposal of Assessment Protocol in candidates to cochlear implant with cerebral palsy from the “Centro de Pesquisas Audiológicas do Hospital de Reabilitação de Anomalias Craniofaciais da USP” in order to establish rules and considerations in evaluation of these children. The pilot study was developed with a boy with neuropathy, profound hearing loss bilateral, quadriparesia atetóide, 2 years old and 7 months, premature, with history of hyperbilirrubinemia and ototoxin, usuary of hearing aids since the 8° month of birth. The protocol consisted: medical, neurological and otorrynolaryngological assessments, audiological assessment (VRA, tympanometry, imitanciometry, OAE transient/distorcion product, EAPs, Stable State Auditory, IT-MAIS, PRISE, Communicative Behavior Observation, MacArthur Inventory, Gesel and Amatruda Scale and ELM Scale (visual and expressive); Psychological assessment (familiar permeability and cognition). The found results were: medical assessments rejected contraindicated for surgery (serious neurological decay, inadequate general health, otitis, cochlear and/or auditory nerve agenesis), normal tympanometry and imitanciometry, OAEt and EAPs absents bilateral, OAEdp presents bilateral, Stable State Auditory present in 110 dB, IT-MAIS: 40%; PRISE: 59%, MacArthur Inventory: predominant communication by gestures; ELM expressive: 3 months, visual compatible with age; Gesel and Amatruda Scale changed for all aspects; cognition and ideal familiar permeability. The detailed assessment of aspects about the global development in a child with hearing impairment and cerebral palsy makes indispensable for the rehabilitation team to plan real marks, besides to inform parents about the prognosis of your son. This patient was considered able to cochlear implant surgery, and the results of pos-implantation will be studied in a soon future.

